# Sex differences in the late first trimester human placenta transcriptome

**DOI:** 10.1186/s13293-018-0165-y

**Published:** 2018-01-15

**Authors:** Tania L. Gonzalez, Tianyanxin Sun, Alexander F. Koeppel, Bora Lee, Erica T. Wang, Charles R. Farber, Stephen S. Rich, Lauren W. Sundheimer, Rae A. Buttle, Yii-Der Ida Chen, Jerome I. Rotter, Stephen D. Turner, John Williams, Mark O. Goodarzi, Margareta D. Pisarska

**Affiliations:** 1 Department of Obstetrics and Gynecology, Division of Reproductive Endocrinology and Infertility, Cedars-Sinai Medical Center, Los Angeles, CA USA; 2 Center for Public Health Genomics, University of Virginia, Charlottesville, VA USA; 3 Division of Reproductive Endocrinology and Infertility, UCLA David Geffen School of Medicine, Los Angeles, CA USA; 4 Department of Obstetrics and Gynecology, Division of Maternal-Fetal Medicine, Cedars-Sinai Medical Center, Los Angeles, CA USA; 5 LABiomed/Harbor-UCLA Medical Center, Torrance, CA USA; 6 Department of Medicine, Division of Endocrinology, Diabetes and Metabolism, Cedars-Sinai Medical Center, Los Angeles, CA USA

**Keywords:** Sex differences, First trimester placenta, Chorionic villus sampling, Pregnancy, RNA-sequencing

## Abstract

**Background:**

Development of the placenta during the late first trimester is critical to ensure normal growth and development of the fetus. Developmental differences in this window such as sex-specific variation are implicated in later placental disease states, yet gene expression at this time is poorly understood.

**Methods:**

RNA-sequencing was performed to characterize the transcriptome of 39 first trimester human placentas using chorionic villi following genetic testing (17 females, 22 males). Gene enrichment analysis was performed to find enriched canonical pathways and gene ontologies in the first trimester. DESeq2 was used to find sexually dimorphic gene expression. Patient demographics were analyzed for sex differences in fetal weight at time of chorionic villus sampling and birth.

**Results:**

RNA-sequencing analyses detected 14,250 expressed genes, with chromosome 19 contributing the greatest proportion (973/2852, 34.1% of chromosome 19 genes) and Y chromosome contributing the least (16/568, 2.8%). Several placenta-enriched genes as well as histone-coding genes were identified to be unique to the first trimester and common to both sexes. Further, we identified 58 genes with significantly different expression between males and females: 25 X-linked, 15 Y-linked, and 18 autosomal genes. Genes that escape X inactivation were highly represented (59.1%) among X-linked genes upregulated in females. Many genes differentially expressed by sex consisted of X/Y gene pairs, suggesting that dosage compensation plays a role in sex differences. These X/Y pairs had roles in parallel, ancient canonical pathways important for eukaryotic cell growth and survival: chromatin modification, transcription, splicing, and translation.

**Conclusions:**

This study is the first characterization of the late first trimester placenta transcriptome, highlighting similarities and differences among the sexes in ongoing human pregnancies resulting in live births. Sexual dimorphism may contribute to pregnancy outcomes, including fetal growth and birth weight, which was seen in our cohort, with males significantly heavier than females at birth. This transcriptome provides a basis for development of early diagnostic tests of placental function that can indicate overall pregnancy heath, fetal-maternal health, and long-term adult health.

**Electronic supplementary material:**

The online version of this article (10.1186/s13293-018-0165-y) contains supplementary material, which is available to authorized users.

## Background

In humans and other placental mammals, the fertilized egg gives rise to both the fetus and the placenta. Placentation in the first trimester can impact fetal growth, and abnormal placentation can lead to more pronounced effects complicating pregnancy including intrauterine growth restriction (IUGR) which results in very low birth weight infants, a sexually dimorphic outcome [1–4]. The process of placentation occurs throughout the first trimester of pregnancy, whereby the outer cells of the blastocyst (the trophoblast cells) invade the maternal tissue and develop into the placenta. It is a highly regulated state of active cell proliferation, cell migration, and cell differentiation [5]. To sustain this growth, placentation is in a high state of transcriptional activity, as evidenced by its marked hypomethylated state [5–9]. Placentation requires multiple factors, including maternal immune tolerance, various growth factors, fetal-maternal communication via chemical signaling, and a receptive maternal decidua that allows extravillous trophoblast cells to invade the maternal circulatory system and access maternal nutrients throughout pregnancy [5, 10–13].

Fetal growth, development, and well-being is impacted by fetal sex [14, 15]. Females are slightly smaller as early as 8–12 weeks and continue to be smaller throughout gestation [16, 17]. Fetal survival rates are also sexually dimorphic in early pregnancy. Male fetuses have a 2.00–2.50-fold higher risk for spontaneous abortions in the first trimester and 1.25-fold higher risk before 23 weeks (mid-second trimester) [18]. Despite studies describing the placental transcriptome at term and differences attributed to fetal sex [19–21], there are no studies looking at the first trimester placenta, which is a critical time during development that may contribute to sexually dimorphic outcomes and is a time when pregnancy complications likely manifest [7, 22]. In addition, it is during this time that origins of common adult diseases such as hypertension, coronary heart disease, type 2 diabetes, and depression may develop, which are also sexually dimorphic [23, 24].

Thus, our goal is to identify the late first trimester placenta transcriptome and identify gene expression differences due to fetal sex. This work provides a valuable reference of the late first trimester placental transcriptome at the earliest time point of placental development that can be studied in ongoing human pregnancies. It can be used to develop a sex-specific placenta atlas unique to humans, important because the human placenta has unique features not found in animals models [25]. In addition, findings from these studies may be the initial step in identifying potential mechanisms in placentation that lead to sexually dimorphic outcomes in fetal growth and development as well as long-term adult health, and can ultimately be used to develop non-invasive diagnostic testing of fetal well-being [26].

## Methods

### Chorionic villi sample procurement

First trimester placenta was obtained from discarded chorionic villi at 10.5–13.5 weeks from chorionic villus sampling (CVS) used for genetic testing, with informed written consent. All protocols were performed in accordance with the institutional review board’s guidelines at the Cedars-Sinai Medical Center under IRB Protocols Pro00006806 (Prenatal repository) and Pro00008600 (differential gene expression of early placenta). Remaining CVS tissue that was not used for genetic testing was cleaned to separate maternal-derived decidua tissue and fetal-derived chorionic villi. Tissue samples (5–15 mg) were kept on ice and submerged in 250 μl RNA*later* RNA Stabilization Reagent (QIAGEN, Hilden, Germany) within 30 min, then stored at − 80 °C in Cedar-Sinai Medical Center’s Prenatal Repository until further processing. All subjects were Caucasian, spontaneous singleton conceptions that resulted in a live birth. There were 17 females and 22 males. All karyotypes were either 46,XX or 46,XY.

### Subject demographics analysis

Subject demographics including maternal age, race, ethnicity, pre-pregnancy BMI, as well as fetal gestational age at CVS, crown-rump length (CRL), karyotype analysis, gestational age at delivery, and birthweight were analyzed for *n* = 39 subjects. Dating by last menstrual period, when available, and early initial ultrasonography was completed using crown-rump length (CRL) measurements to confirm gestational age. Gestational age and CRL measured at the time of CVS were used for analysis. *t* test was used for continuous variables and Fisher’s exact test was used for categorical variables. Eight samples lacked complete clinical data for birthweight, gestational age, or crown-rump length. Omitting those samples (leaving *n* = 31), the results were unchanged.

### RNA extraction from chorionic villi tissue

CVS were processed as previously described [27]. Briefly, CVS tissue was thawed on ice with 600 μl of RTL Plus lysis buffer (QIAGEN) and 1% β-mercaptoethanol added to each sample. Cells were homogenized by passing at least 10 times through single-use needles with decreasing gauge (22G, 25G then 27G) attached to a 1 mL sterile, RNase-free syringe. The homogenates were loaded onto AllPrep spin columns and the remainder of the protocol was performed following manufacturer instructions. DNA and RNA were extracted using the commercial AllPrep DNA/RNA Mini Kit (QIAGEN) for RNA-seq. RNA was eluted with 30 μl of the kit-provided RNase-free water at room temperature, then the elution was passed through the column a second time to increase RNA yield. DNA was eluted with 100 μl of the kit-provided EB buffer warmed to 70 °C. Equal numbers of male and female samples were processed each time to reduce batch effects. The average RNA integrity number (RIN) score for RNA samples was 7.70.

### RNA-sequencing and statistical analysis

RNA-Seq libraries were constructed from 200 ng of total RNA using Illumina TruSeq Stranded Total RNA with Ribo-Zero Gold sample prep kits (Illumina, Carlsbad, CA, USA). Constructed libraries contained RNAs > 200 nt (both unpolyadenylated and polyadenylated) and were depleted of cytoplasmic and mitochondrial rRNAs. An average of 22.53 million 2 × 75 bp paired-end reads were generated for each sample on an Illumina NextSeq 500. Reads were aligned to the human reference genome (build GRCh38 with Ensembl release version 87 and UCSC release 24) using STAR [28] and we counted reads mapping to Ensembl genes using the featureCounts software in the Subread package [29]. We then used the DESeq2 Bioconductor package [30, 31] in the R statistical computing environment [32] to normalize count data, estimate dispersion, and fit a negative binomial model for each gene. The Benjamini-Hochberg false discovery rate (FDR) procedure was used to estimate the adjusted *P* values for Ensembl gene IDs. Significant genes were defined as below 5% FDR.

### Quality control to verify sample identity

To ensure samples did not contain maternal contamination, RNA sequencing results were screened for decidua-specific genes (*SCARA5*, *PIGF*, *SLPI*) and samples excluded if any decidua-specific genes were expressed. Fetal sex, identified on cytogenetic analysis, was also verified after RNA-sequencing using the individual baseMean and fragments per kilobase per million mapped fragments (FPKM) levels of *XIST* (a female-specific gene) and highly expressed Y chromosome genes (*DDX3Y*, *KDM5D*, *UTY*, and *ZFY*).

### Expression threshold selection

In order to identify the transcription profile of the late first trimester human placenta of ongoing pregnancies, an expression threshold of FPKM > 1.281 was selected. After DESeq2 analysis, the mean FPKM from female samples was found for all Y-linked genes. The maximum value was 1.2808 for *CD24P4*, an outlier 13 standard deviations higher than the next value, 0.3968 for *PSMA6P1*. Signal for *CD24P4* was likely sequencing noise from *CD24*, a highly expressed chromosome 6 gene with 99.43% identity to *CD24P4* (BLASTN *E* value = 2.0 × 10^−90^) found using the BLAST tool from the National Center for Biotechnology Information (NCBI) [33]. The higher threshold was selected to ensure a reliable transcriptome atlas. Genes on all chromosomes were considered expressed if they reached mean FPKM > 1.281 in females, males, or both.

### Pathway and gene enrichment analysis

To investigate the biological significance of our gene subset, data were analyzed with Ingenuity Pathways Analysis (IPA) software (QIAGEN, Redwood City, CA, USA, www.qiagenbioinformatics.com/IPA), as previously described [34, 35]. Genes were partitioned into quartiles after sorting by maximum average FPKM (male or female). Gene groups were analyzed for enrichment in gene ontology, canonical pathways, and upstream regulators. A right-tailed Fisher’s exact test was used to calculate *P* values of enriched categories. The chromosome location of upstream regulators was found by cross-referencing gene names from IPA with the db2db tool from biological DataBase network (Frederick National Laboratory for Cancer Research, https://biodbnet-abcc.ncifcrf.gov/db/db2db.php) [36]. The top 25% of expressed genes were input into IPA for upstream analysis and 98.0% (3493 of 3563) were accepted. Upstream regulators were ranked by most significant *P* value (Fisher’s exact test). Full names and descriptions were pulled from the National Institute of Health NCBI databases (Gene, PubChem) and miRbase: the microRNA database [33, 37]. For each upstream regulator, the percent of accepted input genes that it affects is shown as a percentage (e.g., 195/3493 × 100% for D-glucose) in column “% Genes Regulated.”

### Identification of placental specific or enriched transcripts

In order to identify highly expressed genes with placenta tissue-specificity, we performed an in silico analysis of tissue specificity on genes with FPKM > 128. Protein-coding genes were ranked by highest FPKM value across all samples in the first trimester. We referenced the expressed sequence tag (EST) profile breakdown by healthy body sites (typically 45 sites) of protein-coding genes using the NCBI UniGene database (https://www.ncbi.nlm.nih.gov/unigene) accessed 15 Aug 2017 [33]. To determine placenta enrichment, the transcripts per million (TPM) of protein-coding genes in different normal body sites were compared. Genes with no placenta expression in UniGene (TPM = 0) were validated to be FPKM < 2 in term placenta using RNA-seq data from NCBI Gene Expression Omnibus (GEO) database, accession GSE73016 [38].

### Venn diagram of gene ontology for sexually dimorphic genes

The Venn diagram for differentially expressed genes (DEGs) was created with modifications from Bellott et al. [39]. Additional functional categories were added in an effort to maximize the number of sexually dimorphic genes represented. The NCBI Gene database (https://www.ncbi.nlm.nih.gov/gene) and UniProt [40] were used for an initial ontology scan then PubMed was cross-referenced to verify gene ontology (https://www.ncbi.nlm.nih.gov/pubmed/). Almost all DEGs (43/58; 74.2%) had known or predicted function.

### Overview of X chromosome inactivation status and X/Y gene homology

The human genome annotation file (GRCh38) was downloaded from Ensembl [41]. The annotations for the 25 differentially expressed X-linked genes (FDR < 0.05) were extracted from the human genome annotation file and input to NCBI Genome Decoration Page (https://www.ncbi.nlm.nih.gov/genome/tools/gdp) to generate the ideogram of the X chromosome showing the locations of these genes. The consensus calls for X inactivation status were adapted from Balaton et al. [42], which compiled a comprehensive list of X inactivation statuses from several studies. Genes that were reported to be subject to X inactivation in most or all of these studies were categorized as X inactivated genes (color-coded red). Genes that were reported to be escaping X inactivation in most or all of these studies were categorized here as escaping genes (color-coded blue), including one gene (*CHM*) that had an even split of X chromosome inactivation (XCI) status calls between studies. On the human X chromosome, 25% of X-linked genes located outside of pseudo-autosomal regions have Y counterparts [42], which are either functional (“Y homologs”) or non-functional (“Y pseudogenes”) [39, 43]. Table 7 listed all X/Y gene pairs that contain functional Y homologs, but not non-functional Y pseudogenes. All X-linked genes listed in Table 7 are protein-coding genes.

## Results

### Identification of a transcriptome signature from late first trimester human placenta

**Fig. 1:**
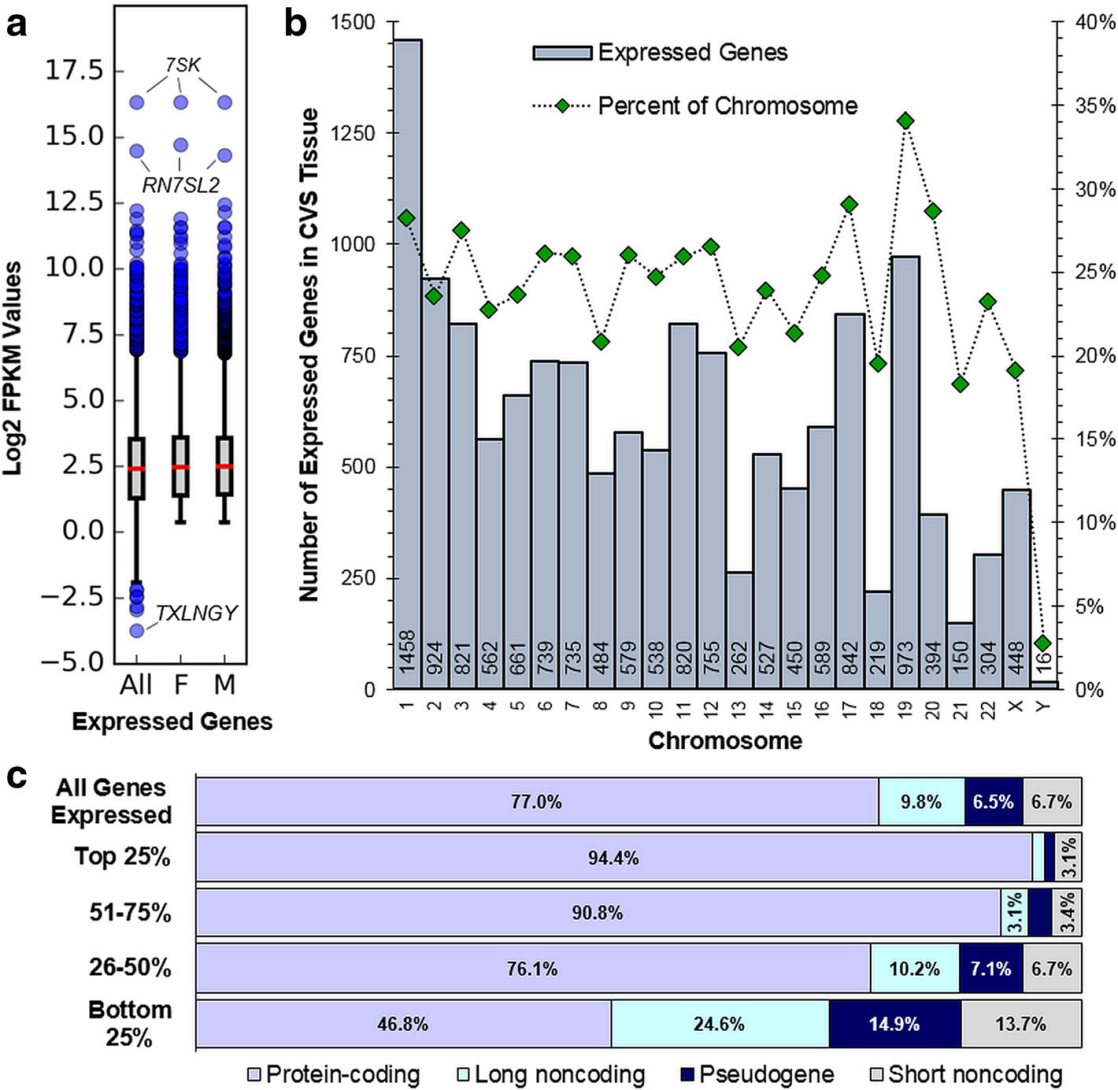
Normative late first trimester placenta transcriptome. **a** Box and whisker plot showing log_2_FPKM distribution of expressed genes. All: average log_2_FPKM of 14,250 expressed genes over all 39 samples. F, M: all genes over the FPKM cutoff in either female or male samples, respectively. The whiskers range is the interquartile range ± 50%, with outliers shown as blue circles. Median is red line. **b** Chromosome distribution of 14,250 genes expressed in chorionic villi, all biotypes (gray bars). Expressed genes are shows as a percentage of total genes in each chromosome (green diamonds). **c** Biotype categories of all expressed genes and each FPKM quartile are shown. The proportion of each biotype is shown as a percentage and labeled if ≥ 3%

To identify the transcriptional profile of the late first trimester human placenta, RNA sequencing was performed on chorionic villi from 39 singleton pregnancies (17 female and 22 male samples). RNA-sequencing resulted in approximately 22.53 million 2 × 75 bp pair-ended reads per sample. Genes were annotated using human reference build GRCh38. Out of 57,543 human genes, a total of 47,778 genes had non-zero baseMean values indicating possible detection in chorionic villi. Principal components analysis showed clusters separated by sex (Additional file 1A). To determine whether each gene was expressed or not in chorionic villi regardless of sex, the FPKM values were used to identify a normative first trimester placental signature. An FPKM > 1.281 threshold was selected to remove sequencing noise, higher than the typical arbitrary threshold of FPKM > 1. This higher threshold was selected to remove all background sequencing noise which produced low levels of Y chromosome signal from cytogenetically confirmed female samples (no Y chromosome). A total of 14,250 genes were determined to comprise the normative first-trimester placenta transcriptome: 13,319 present in both males and females, and the remaining 931 genes above the expression threshold in one sex but not the other. The log_2_FPKM of expressed genes, as an average of all samples, ranged from − 3.7356 to 16.3245 (Fig. 1a).

The chromosomal distribution of the expressed genes (protein-coding, long non-coding, pseudogenes, and short non-coding) are presented in Fig. 1b. The highest number of expressed genes is on chromosome 1 followed by one of the shortest chromosomes, chromosome 19 (Fig. 1b). Since gene distribution is not proportional to chromosome size, we looked at the proportion of expressed genes in the placenta as a percentage of total genes for each chromosome using human genome assembly GRCh38.p7 [41]. Even after controlling for gene distribution, 34.1% (973/2852) of genes on chromosome 19 were expressed, the highest of any other chromosome. The Y chromosome had the lowest proportion of expressed genes with only 2.8% (16 genes) of all 568 Y chromosome genes annotated in human genome assembly GRCh38.p7. Of these 16 Y-linked genes, there were 8 protein-coding, 4 long non-coding, and 4 pseudogenes.

### Expressed genes in the late first trimester human placenta

The biotypes of all expressed transcripts of the normative late first trimester placenta transcriptome consist of 77.0% protein-coding genes, 9.8% long non-coding genes, 6.5% pseudogenes, and 6.7% short non-coding genes (Fig. 1c). Expressed genes were sorted by highest-to-lowest FPKM values using the female average or male average for each gene (whichever was higher) and separated into quartiles of approximately 3562 genes each. This method best represented the high expression of sex-specific genes such as Y-linked genes absent in females*,* which would otherwise drop from the highest quartile to the lowest quartile if the average FPKM of all 39 samples were used. The biotype distributions of the top two quartiles were similar in the first trimester placenta. Within the highest quartile (top 25% expressed genes), 94.4% (3364) were protein-coding genes, 1.3% (48) were long non-coding, 1.1% (40) were pseudogenes, and 3.1% (111) were short non-coding (Fig. 1c, Additional file 2). Of the short non-coding genes in the highest quartile, 32.4% (36 of 111) were microRNA-encoding genes, of which 66.7% (24 of 36) were located on chromosome 19. Within the second highest quartile (51-75% of sorted genes), 90.8% (3234) were protein-coding, 3.1% (111) were long non-coding, 2.7% (97) were pseudogenes, and 3.4% (120) were short non-coding genes (Fig. 1c). The proportion of non-coding genes only reached > 10% in the lower two quartiles (Fig. 1c).

Ingenuity Pathways Analysis (IPA) for gene enrichment analysis was performed for expressed genes in the first trimester placenta, partitioned into quartiles (Table 1). In the most highly expressed quartile, the enriched canonical pathways were EIF2 signaling, regulation of eIF4 and p70S6K signaling, protein ubiquitination, mitochondrial dysfunction, and sirtuin signaling. These pathways are essential for protein synthesis, cell growth, and energy metabolism. Within the second highest quartile, the most enriched canonical pathways were related to hormonal signaling and growth regulation, including estrogen, JAK/STAT, ceramide, insulin, and STAT3 signaling.

**Table 1:** Enriched canonical pathways for expressed genes in the late first trimester placenta, by FPKM quartiles

Input	Top canonical pathways	*P* value^a^	# genes
Top 25% of genes	EIF2 signaling	8.25E-55	136
	Regulation of eIF4 and p70S6K signaling	7.88E-26	81
	Protein ubiquitination pathway	5.90E-25	111
	Mitochondrial dysfunction	1.07E-22	81
	Sirtuin signaling pathway	5.43E-20	109
51–75% quartile	Estrogen receptor signaling	2.12E-10	48
	JAK/STAT signaling	9.83E-08	32
	Ceramide signaling	1.80E-07	34
	Insulin receptor signaling	2.18E-07	45
	STAT3 pathway	2.57E-07	29
26–50% quartile	Melanocyte development and pigmentation signaling	7.85E-04	24
	NGF signaling	7.96E-04	28
	ERK/MAPK signaling	1.59E-03	40
	HGF signaling	1.72E-03	26
	Acyl-CoA hydrolysis	1.76E-03	6
0–25% quartile	PI3K signaling in B lymphocytes	9.40E-04	21
	CDP-diacylglycerol biosynthesis I	1.71E-03	7
	Wnt/Ca^2+^ pathway	2.81E-03	12
	Phosphatidylglycerol biosynthesis II (non-plastidic)	2.83E-03	7
	D-myo-inositol-5-phosphate metabolism	3.20E-03	23

^a^Fisher’s exact test

Among the top 25% most expressed genes, the most enriched molecular and cellular functions were cell death and survival, protein synthesis, cellular development, cell growth and proliferation, and cellular movement (Table 2). The top five enriched categories for physiological systems development and function were organismal survival, cardiovascular systems development and function, connective tissue development and function, tissue development, and organismal development (Table 2). Upstream analysis in IPA identified the top 40 gene regulators upstream of the top 25% expressed genes (Additional file 3). Most upstream regulators were associated with either essential cell regulation, cell growth, or hormonal signaling. Transcriptional regulators such as MYC (*P* = 1.70 × 10^−70^), p53 (*P* = 1.63 × 10^−65^), MYCN (*P* = 3.17 × 10^−63^), and HNF4A (*P* = 2.10 × 10^−58^) were among the most significant upstream regulators (Additional file 3). Regulators involved in cell growth include rapamycin-insensitive companion of mTOR (*RICTOR*), transforming growth factor beta 1 (*TGFB1*), D-glucose, epidermal growth factor receptor (*EGFR*), as well as epidermal growth factor (*EGF*). In addition, hormones such as beta-estradiol, its receptor (*ESR1*), as well as the progesterone receptor (*PGR*) were also significant upstream regulators. Together, these upstream regulators control essential cell regulation, cell growth, and hormonal signaling, consistent with the canonical pathways identified (Additional file 3).

**Table 2:** Enriched gene ontology for top 25% of expressed genes

	*P* value range^a^	# genes
Molecular and cellular function		
Cell death and survival	7.97E-11 to 2.76E-96	1356
Protein synthesis	3.01E-11 to 8.66E-70	598
Cellular development	3.61E-11 to 1.31E-57	999
Cellular growth and proliferation	3.61E-11 to 1.31E-57	953
Cellular movement	1.05E-10 to 6.29E-53	892
Physiological systems development and function		
Organismal survival	1.31E-15 to 1.52E-63	958
Cardiovascular system development and function	9.45E-11 to 1.20E-31	576
Connective tissue development and function	8.86E-11 to 4.80E-27	579
Tissue development	7.01E-11 to 4.80E-27	747
Organismal development	9.45E-11 to 3.54E-23	1008

^a^Fisher’s exact test for sub-categories from IPA shown as *P* value range

In order to identify highly expressed genes with placenta-enriched tissue expression, we selected protein-coding genes with FPKM > 128, two orders of magnitude above the expression threshold followed by an in silico assessment that compared them to EST profiles of healthy human tissue in the NCBI UniGene database (http://www.ncbi.nlm.nih.gov/unigene/) (Table 3). There were 175 protein-coding genes with FPKM > 128 in the first trimester placenta, with the highest expression from *KISS1* (metastasis-suppressor), *CGA* (glycoprotein hormones alpha subunit), *TFPI2* (tissue factor pathway inhibitor 2), *CSH1* and *CSH2* (chorionic somatomammotropin hormones), *CGB8 and CGB5* (chorionic gonadotropin beta subunits), *SPP1* (secreted phosphoprotein 1), *TMSB10* (thymosin beta 10), and *EEF1A1* (eukaryotic translation elongation factor 1 alpha 1). Of these 175 protein-coding genes, 35 (20%) were known to be placenta-enriched, with UniGene-documented transcripts per million (TPM) highest in placenta compared to other tissues (Table 3). Two genes, *XAGE3* and *HIST1H2BI*, were exclusively expressed in the placenta, with *HIST1H2BI* showing TPM = 0 in all healthy tissue on UniGene (Table 3). Sixteen genes (*CGB3*, *CGB5*, *CGB8*, *HIST1H1B*, *HIST1H1E*, *HIST1H2BO*, *HIST1H3B*, *HIST1H3C*, *HIST1H3F*, *HIST1H4B*, *HIST1H4D*, *HIST1H4F*, *PSG3*, *PSG6*, *PSG9*, and *XAGE2*) were almost specific to the placenta, with expression in 6 other tissues or less, most of embryonic or vascular system origin. Thirteen genes (*EBI3*, *HIST1H1B*, *HIST1H1D*, *HIST1H1E*, *HIST1H2BI*, *HIST1H2BO*, *HIST1H3B*, *HIST1H3C*, *HIST1H3F*, *HIST1H4B*, *HIST1H4C*, *HIST1H4D*, and *HIST1H4F*) had no UniGene-documented placenta tissue expression, although some were expressed in many other tissues (Table 3).

**Table 3:** Highest expressed protein-coding genes in late first trimester placenta

#	Gene symbol	Chr	Description	TPM highest in placenta?^a^	Placenta specific?^b^	Where else expressed?^b^	Upregulated in first vs term^c^
1	KISS1	1	KiSS-1 metastasis-suppressor	+ + +	N		Term
2	CGA	6	Glycoprotein hormones, alpha polypeptide	N	N		
3	TFPI2	7	Tissue factor pathway inhibitor 2	N	N		
4	CSH1	17	Chorionic somatomammotropin hormone 1	+ +	N		
5	CSH2	17	Chorionic somatomammotropin hormone 2	+	N		
6	CGB8	19	Chorionic gonadotropin beta subunit 8	+ +	Almost	Embryonic tissue, skin	
7	CGB5	19	Chorionic gonadotropin beta subunit 5	+ +	Almost	Embryonic tissue, testis, heart, skin	
8	SPP1	4	Secreted phosphoprotein 1	N	N		
9	TMSB10	2	Thymosin beta 10	N	N		
10	EEF1A1	6	Eukaryotic translation elongation factor 1 alpha 1	N	N		
11	PEG10	7	Paternally expressed 10	N	N		
12	FTL	19	Ferritin light chain	N	N		
13	CGB3	19	Chorionic gonadotropin beta subunit 3	+ +	Almost	Embryonic tissue, connective tissue, testis, ovary	
14	RPS12	6	Ribosomal protein S12	N	N		
15	PSG3	19	Pregnancy specific beta-1-glycoprotein 3	+ + +	Almost	Vascular, testis, muscle, kidney, intestine, skin	Term
16	ACTB	7	Actin beta	N	N		
17	IFI6	1	Interferon alpha inducible protein 6	N	N		
18	COL3A1	2	Collagen type III alpha 1 chain	N	N		
19	GDF15	19	Growth differentiation factor 15	+ +	N		Term
20	PAGE4	X	PAGE family member 4	+	N	Prostate, heart	
21	RPS27	1	Ribosomal protein S27	N	N		
22	ACTG1	17	Actin gamma 1	N	N		
23	FN1	2	Fibronectin 1	N	N		
24	RPL13A	19	Ribosomal protein L13a	N	N		
25	HIST1H1C	6	Histone cluster 1 H1 family member c	N	N		
26	RPS11	19	Ribosomal protein S11	N	N		
27	S100A11	1	S100 calcium binding protein A11	N	N		
28	HIST1H2BK	6	Histone cluster 1 H2B family member k	N	N		
29	HIST1H3B	6	Histone cluster 1 H3 family member b	No value	Almost	Thymus, liver, kidney, testis	
30	HIST1H1E	6	Histone cluster 1 H1 family member e	No value	Almost	Mouth, kidney, brain	
31	PSAP	10	Prosaposin	N	N		
32	EEF2	19	Eukaryotic translation elongation factor 2	N	N		
33	RPS18	6	Ribosomal protein S18	N	N		
34	RPL10A	6	Ribosomal protein L10a	N	N		
35	CALR	19	Calreticulin	N	N		
36	HIST1H1B	6	Histone cluster 1 H1 family member b	No value	Almost	Bladder, intestine, skin	
37	TGM2	20	Transglutaminase 2	N	N		
38	RPL7A	9	Ribosomal protein L7a	N	N		
39	SAT1	X	Spermidine/spermine N1-acetyltransferase 1	N	N		
40	XAGE3	X	X antigen family member 3	+	Yes		
41	HIST1H4D	6	Histone cluster 1 H4 family member d	No value	Almost	Adipose tissue, vascular	
42	HSPA8	11	Heat shock protein family A (Hsp70) member 8	N	N		
43	PSG4	19	Pregnancy specific beta-1-glycoprotein 4	+ +	N		Term
44	GAPDH	12	Glyceraldehyde-3-phosphate dehydrogenase	N	N		
45	EBI3	19	Epstein-Barr virus induced 3	No value*	N		Term
46	RPS4X	X	Ribosomal protein S4, X-linked	N	N		
47	RPS6	9	Ribosomal protein S6	N	N		
48	RPS8	1	Ribosomal protein S8	N	N		
49	EFEMP1	2	EGF containing fibulin-like extracellular matrix protein 1	N	N		
50	RPLP0	12	Ribosomal protein lateral stalk subunit P0	N	N		
51	ADAM12	10	ADAM metallopeptidase domain 12	+ +	N		Term
52	S100A9	1	S100 calcium binding protein A9	N	N		Term
53	CYP19A1	15	Cytochrome P450 family 19 subfamily A member 1	+ + +	N		Term
54	COL4A1	13	Collagen type IV alpha 1 chain	N	N		
55	ANXA5	4	Annexin A5	N	N		
56	RPL19	17	Ribosomal protein L19	N	N		
57	KRT18	12	Keratin 18	N	N		
58	XAGE2	X	X antigen family member 2	+ +	Almost	Heart, uterus	
59	HSP90AB1	6	Heat shock protein 90 alpha family class B member 1	N	N		
60	HIST1H4C	6	Histone cluster 1 H4 family member c	No value	N		
61	IGF2	11	Insulin-like growth factor 2	N	N		
62	HSD3B1	1	Hydroxy-delta-5-steroid dehydrogenase, 3 beta- and steroid delta-isomerase 1	+ +	N		
63	EZR	6	Ezrin	N	N		
64	SLC2A1	1	Solute carrier family 2 member 1	N	N		Term
65	RPS2	16	Ribosomal protein S2	N	N		
66	FBLN1	22	Fibulin 1	+	N		Term
67	S100P	4	S100 calcium binding protein P	N	N		Term
68	SDC1	2	Syndecan 1	N	N		Term
69	HIST1H3F	6	Histone cluster 1 H3 family member f	No value	Almost	Liver, connective tissue, skin	
70	CTSL	9	Cathepsin L	N	N		
71	HIST1H4F	6	Histone cluster 1 H4 family member f	No value	Almost	Blood, connective tissue	
72	PSG1	19	Pregnancy specific beta-1-glycoprotein 1	+ + +	N		Term
73	RPS25	11	Ribosomal protein S25	N	N		
74	PSG5	19	Pregnancy specific beta-1-glycoprotein 5	+ +	N		
75	PLAC4	21	Placenta specific 4	+ + +	N		
76	RPL11	1	Ribosomal protein L11	N	N		
77	SPARC	5	Secreted protein acidic and cysteine rich	N	N		
78	KRT19	17	Keratin 19	N	N		
79	PSG2	19	Pregnancy specific beta-1-glycoprotein 2	+ + +	N		
80	RPL8	8	Ribosomal protein L8	N	N		
81	HSP90B1	12	Heat shock protein 90 beta family member 1	N	N		
82	GPC3	X	Glypican 3	N	N		
83	RPL7	8	Ribosomal protein L7	N	N		
84	PAPPA2	1	Pappalysin 2	+ +	N		Term
85	KRT8	12	Keratin 8	+	N		
86	RPL5	1	Ribosomal protein L5	N	N		
87	GH2	17	Growth hormone 2	+	N		
88	MEST	7	Mesoderm specific transcript	N	N		
89	RPN2	20	Ribophorin II	N	N		
90	PRDX5	11	Peroxiredoxin 5	N	N		
91	GSTP1	11	Glutathione S-transferase pi 1	N	N		
92	LDHB	12	Lactate dehydrogenase B	N	N		
93	HIST1H3C	6	Histone cluster 1 H3 family member c	No value	Almost	Testis	
94	VGLL1	X	Vestigial-like family member 1	+ +	N		
95	RPL4	15	Ribosomal protein L4	N	N		
96	ENO1	1	Enolase 1	N	N		First
97	PAPPA	9	Pappalysin 1	+ +	N		Term
98	COL4A2	13	Collagen type IV alpha 2 chain	N	N		
99	RPS3A	4	Ribosomal protein S3A	N	N		
100	SLC40A1	2	Solute carrier family 40 member 1	N	N		
101	TIMP3	22	TIMP metallopeptidase inhibitor 3	N	N		
102	ANXA1	9	Annexin A1	N	N		
103	RPL35	9	Ribosomal protein L35	N	N		
104	TPT1	13	Tumor protein, translationally controlled 1	N	N		
105	EPAS1	2	Endothelial PAS domain protein 1	N	N		
106	HIST1H2BI	6	Histone cluster 1 H2B family member i	No value	Yes	No value in any UniGene body site	
107	S100A6	1	S100 calcium binding protein A6	N	N		
108	RPL3	22	Ribosomal protein L3	N	N		
109	RPL12	9	Ribosomal protein L12	N	N		
110	DUSP9	X	Dual specificity phosphatase 9	N	N		
111	PSG9	19	Pregnancy specific beta-1-lycoprotein 9	+ + +	Almost	Vascular, spleen, eye,mammary gland, lung, skin	Term
112	FOXO4	X	Forkhead box O4	+ +	N		
113	PEG3	19	Paternally expressed 3	N	N		
114	RPL41	12	Ribosomal protein L41	N	N		
115	FAM129B	9	Family with sequence similarity 129 member B	N	N		
116	PDIA3	15	Protein disulfide isomerase family A member 3	N	N		
117	HIST1H4B	6	Histone cluster 1 H4 family member b	No value	Almost	Lymph node, testis, liver	
118	MYL12B	18	Myosin light chain 12B	N	N		
119	HIST1H1D	6	Histone cluster 1 H1 family member d	No value	N		
120	SPINT1	15	Serine peptidase inhibitor, Kunitz type 1	+	N		
121	RPLP2	11	Ribosomal protein lateral stalk subunit P2	N	N		
122	FBN2	5	Fibrillin 2	+	N		
123	RPS27A	2	Ribosomal protein S27a	N	N		
124	BSG	19	Basigin (Ok blood group)	N	N		
125	CTNNB1	3	Catenin beta 1	N	N		
126	RPL21	13	Ribosomal protein L21	N	N		
127	COL6A2	21	Collagen type VI alpha 2 chain	N	N		First
128	RASA1	5	RAS p21 protein activator 1	+ +	N		
129	ATP5B	12	ATP synthase, H+ transporting, mitochondrial F1 complex, beta polypeptide	N	N		
130	RPS20	8	Ribosomal protein S20	N	N		
131	RPS13	11	Ribosomal protein S13	N	N		
132	CANX	5	Calnexin	N	N		
133	ISM2	14	Isthmin 2	+ + +	N		
134	RPL9	4	Ribosomal protein L9	N	N		
135	HBG2	11	Hemoglobin subunit gamma 2	N	N		
136	RACK1	5	Receptor for activated C kinase 1	N	N		
137	LUM	12	Lumican	N	N		
138	NPM1	5	Nucleophosmin	N	N		
139	HIST1H2BO	6	Histone cluster 1 H2B family member o	No value*	Almost	Liver	
140	PEBP1	12	Phosphatidylethanolamine binding protein 1	N	N		
141	RPL10	X	Ribosomal protein L10	N	N		
142	P4HB	17	Prolyl 4-hydroxylase subunit beta	N	N		
143	IGFBP3	7	Insulin-like growth factor binding protein 3	N	N		
144	EIF4G2	11	Eukaryotic translation initiation factor 4 gamma 2	N	N		
145	CD63	12	CD63 molecule	N	N		
146	PRDX1	1	Peroxiredoxin 1	N	N		
147	CD24	6	CD24 molecule	N	N		First
148	YBX1	1	Y-box binding protein 1	N	N		
149	BEX3	X	Brain expressed X-linked 3	N	N		
150	HSPA5	9	Heat shock protein family A (Hsp70) member 5	N	N		
151	UBC	12	Ubiquitin C	N	N		
152	MBNL3	X	Muscleblind-like splicing regulator 3	+	N		
153	RPS19	19	Ribosomal protein S19	N	N		
154	RPS14	5	Ribosomal protein S14	N	N		
155	PFN1	17	Profilin 1	N	N		
156	RHOA	3	Ras homolog family member A	N	N		
157	HIST1H2BD	6	Histone cluster 1 H2B family member d	N	N		
158	COX8A	11	Cytochrome c oxidase subunit 8A	N	N		
159	TCEAL9	X	Transcription elongation factor A like 9	N	N		
160	TMSB4X	X	Thymosin beta 4, X-linked	N	N		
161	HSPB8	12	Heat shock protein family B (small) member 8	+	N		Term
162	KRT7	12	Keratin 7	N	N		First
163	TINAGL1	1	Tubulointerstitial nephritis antigen-like 1	+	N		
164	RPL27	17	Ribosomal protein L27	N	N		
165	HSP90AA1	14	Heat shock protein 90 alpha family class A member 1	N	N		
166	HBA2	16	Hemoglobin subunit alpha 2	N			
167	RRBP1	20	Ribosome binding protein 1	N			
168	HMGA1	6	High mobility group AT-hook 1	N	N		First
169	TPI1	12	Triosephosphate isomerase 1	N	N		
170	PTMA	2	Prothymosin, alpha	N	N		
171	RPSA	3	Ribosomal protein SA	N	N		
172	CHCHD2	7	Coiled-coil-helix-coiled-coil-helix domain containing 2	N	N		
173	RPL6	12	Ribosomal protein L6	N	N		
174	PSG6	19	Pregnancy specific beta-1-glycoprotein 6	+ +	Almost	Vascular, heart, skin, intestine	Term
175	UBB	17	Ubiquitin B	N	N		

^a^Placenta enrichment: (+) = placenta had the highest TPM of any body site on UniGene; (++) = placenta TPM value was one order of magnitude higher than the next-highest body site; (+++) = placenta TPM value was two orders of magnitude higher*;* N = placenta is not highest; No value = means placenta TPM = 0 on UniGene. *Validation with GSE73016 found expression in term placenta [38]

^b^Placenta specificity: “specific” was strictly defined as no expression (TPM = 0) in other healthy body sites on UniGene. “Almost” specific was defined as TPM > 0 in six healthy body sites or less (listed). “N” means gene is expressed in at least seven other tissues (not listed)

^c^DEGs between early first trimester (“First”, 45–59 days) and term ("Term") placenta based on microarray studies in Mikheev et al. [44]

To validate this potentially novel first trimester placenta expression, we cross-referenced RNA-sequencing data of term placentas (NCBI GEO Accession GSE73016) and found that *EBI3* and *HIST1H2BO* were expressed in term placenta [38]. We also compared the top 175 protein-coding genes to a microarray study that compared early first trimester placenta (45–59 days) versus C-section delivered term placentas (NCBI GEO Accession GSE9984) and found 23 genes that are differentially expressed between early pregnancy and delivery [44]. Of the top 175 genes, 5 were significantly upregulated in first trimester (*CD24*, *COL6A2*, *ENO1*, *HMGA1*, *KRT7*) and 18 genes were significantly upregulated in term placenta (*ADAM12*, *CYP19A1*, *EBI3*, *FBLN1*, *GDF15*, *HSPB8*, *KISS1*, *PAPPA*, *PAPPA2*, *PSG1*, *PSG3*, *PSG4*, *PSG6*, *PSG9*, *S100A9*, *S100P*, *SDC1*, *SLC2A1*) [44]. The *XAGE2*, *XAGE3*, *CGB* family, and histone-encoding genes were not identified as gestationally different. Overall, there remained 11 histone-encoding genes highly expressed in late first trimester placenta that showed no UniGene-documented placenta expression (Table 3) nor expression in term placenta [38, 45], suggesting they are unique to first trimester placenta and potentially critical for early placentation.

### Sex differences exist in placenta gene expression

Among the 39 first trimester placenta evaluated, there were 17 females and 22 males. There was no statistically significant difference in maternal age, maternal race, maternal ethnicity, pre-pregnancy body mass index (BMI), gestational age at CVS, or gestational age at delivery (Table 4). Mean CRL was 58.8 ± 12.8 mm and 51.8 ± 10.2 mm (*P* = 0.0714) for females and males, respectively. Thus, there was no difference in fetal size in the late first trimester among the sexes, based on CRL, adjusted or unadjusted. There were also no statistically significant differences in second trimester parameters. However, there was a significant difference in the birth weight among the sexes, with males being heavier at 3715 ± 407 g versus 3176 ± 405 g for females (*P* = 0.0006) (Table 4). Even in a multivariate linear regression after adjustment for maternal age, gestational age at delivery and pre-pregnancy BMI, male infants were 401 g heavier (*P* = 0.003, 95% CI 146–657) than female infants at birth (Table 4). This sexual dimorphism in birth weight is consistent with existing literature [15].

**Table 4:** Subject demographics analysis

Demographics of patient samples for RNA-Seq	Female infants (*n* = 17)	Male infants (*n* = 22)	*P* value	*n*
Maternal age, years	39.4 ± 1.8	39.4 ± 2.9	0.9974^a^	39
Maternal race: Caucasian	17 (100%)	22 (100%)	N/A	39
Maternal ethnicity: Hispanic	2 (12%)	3 (14%)	1^b^	39
Maternal pre-pregnancy BMI, kg/m^2^	22.7 ± 3.5	21.9 ± 2.8	0.4416^a^	39
Gestational age at CVS, days	86 ± 7	81 ± 6	0.0503^a^	39
Crown-rump length (CRL), mm	58.8 ± 12.8	51.8 ± 10.2	0.0714^a^	37
Normal karyotype at CVS	17 (100%)	22 (100%)	N/A	39
Gestational age at delivery, days	272 ± 10	276 ± 11	0.1958^a^	37
Birth weight, grams	3176 ± 405	3715 ± 407	0.0006^a^*	34

*Significant

^a^Student’s *t* test

^b^Fisher’s exact test

**Fig. 2:**
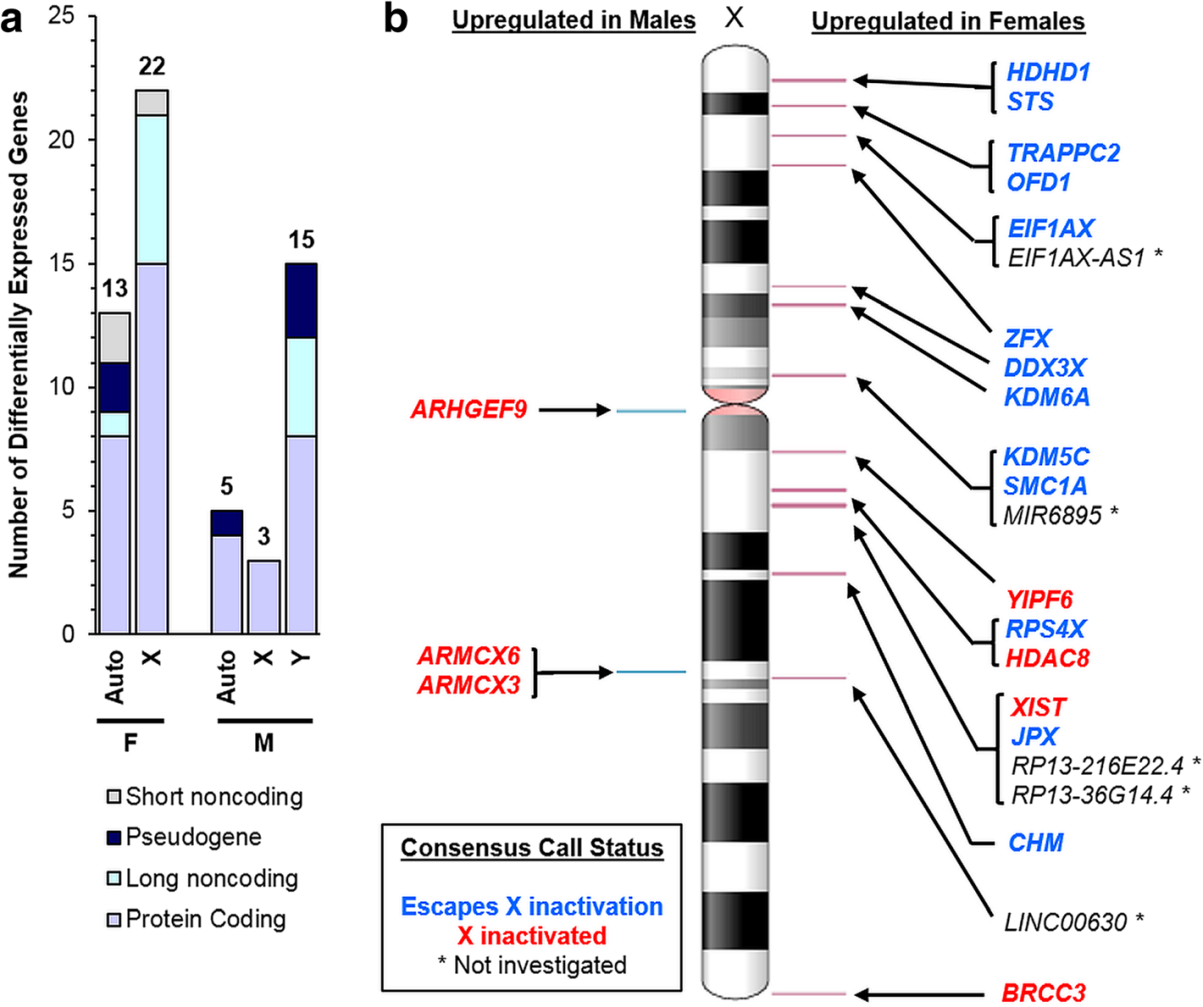
Significantly differentially expressed genes in placenta. **a** Chromosome and biotype distribution of the 58 DEGs, 35 upregulated in females (F) and 23 upregulated in males (M). “Auto” = autosomal chromosomes. **b** Ideogram of chromosome X showing the 25 significantly differentially expressed X-linked genes between first trimester male and female placentas (FDR < 0.05). Left: 3 genes upregulated in males. Right: 22 genes upregulated in females. The consensus calls for X inactivation status were adapted from Balaton et al. [42]

Principal components analysis demonstrated that male and female samples separate into two clusters along a diagonal between the first and second principal components (Additional file 1A). Using DESeq2 on the unfiltered RNA-seq data, volcano and MA plots display the separation of RNA-seq results, with the largest fold-changes in expression coming from Y-linked genes as expected then X-linked genes (Additional file 1B, C). We identified 112 genes significantly different between males and females after adjusting for multiple comparisons and filtering by Benjamini-Hochberg false discovery rate, FDR < 0.05 (Additional file 1 B and Additional file 4). Cell markers for different trophoblasts cell types were not significantly different, suggesting that male and female CVS samples contained similar cell types (Additional file 5). Of 112 genes, 58 genes also meet our FPKM > 1.281 selection criteria, leaving 35 expressed genes significantly upregulated in females and 23 expressed genes significantly upregulated in males (Fig. 2a**,** Tables 5 and 6).

**Table 5:** Genes significantly upregulated in females (downregulated in males)

Gene symbol	Chr	Description	Fold-change (F/M)	FDR^a^	FPKM in F	FPKM in M
XIST	X	X inactive specific transcript (non-protein coding)	26.909	1.83E-172	21.68	0.06
KDM6A	X	Lysine demethylase 6A	1.674	1.36E-52	11.71	6.53
PUDP	X	Pseudouridine 5′-phosphatase	1.653	1.08E-21	12.47	7.04
EIF1AX-AS1	X	EIF1AX antisense RNA 1	1.598	0.00204	2.84	1.34
MIR6895	X	MicroRNA 6895	1.589	0.0353	2.25	0.63
ZNF300	5	Zinc finger protein 300	1.580	0.0257	1.44	0.60
RPL23AP11	14	Ribosomal protein L23a pseudogene 11	1.567	0.0302	2.40	1.27
Y_RNA	5	Y RNA	1.564	0.0438	7.13	3.63
MIR548B	6	MicroRNA 548b	1.552	0.0257	2.00	0.34
EIF1AX	X	Eukaryotic translation initiation factor 1A, X-linked	1.539	4.17E-10	20.05	11.57
RP13-216E22.4	X	[No description]	1.538	0.000323	4.32	2.57
IQCJ-SCHIP1-AS1	3	IQCJ-SCHIP1 read through antisense RNA 1	1.518	0.0105	2.94	1.80
STS	X	Steroid sulfatase (microsomal), isozyme S	1.515	3.89E-05	93.88	57.49
RPS4X	X	Ribosomal protein S4, X-linked	1.490	8.09E-09	336.10	209.62
RP13-36G14.4	X	[no description]	1.471	0.0481	1.64	1.10
DDX3X	X	DEAD-box helicase 3, X-linked	1.434	3.65E-18	74.41	47.42
ITGB8	7	Integrin subunit beta 8	1.402	0.0372	7.96	5.14
ZFX	X	Zinc finger protein, X-linked	1.392	1.81E-07	19.33	12.77
RAB30	11	RAB30, member RAS oncogene family	1.385	0.0494	2.18	1.37
JPX	X	JPX transcript, XIST activator (non-protein coding)	1.379	4.32E-08	5.40	3.62
KDM5C	X	Lysine demethylase 5C	1.364	0.00728	24.18	15.82
SMC1A	X	Structural maintenance of chromosomes 1A	1.360	0.000196	14.72	9.70
TRAPPC2	X	Trafficking protein particle complex 2	1.358	0.000204	4.40	2.97
TBC1D32	6	TBC1 domain family member 32	1.349	0.00774	3.74	2.56
LINC00643	14	Long intergenic non-protein coding RNA 643	1.337	0.0416	6.02	4.01
RASSF6	4	Ras association domain family member 6	1.310	0.00516	12.23	8.74
LINC00630	X	Long intergenic non-protein coding RNA 630	1.295	0.0112	2.92	2.13
BRCC3	X	BRCA1/BRCA2-containing complex subunit 3	1.291	0.000701	10.84	7.50
CHM	X	CHM, Rab escort protein 1	1.269	0.0122	4.49	3.23
YIPF6	X	Yip1 domain family member 6	1.267	0.0209	15.97	11.67
ZNF799	19	Zinc finger protein 799	1.261	0.0172	4.33	3.26
HDAC8	X	Histone deacetylase 8	1.260	0.000121	3.96	2.92
PHTF1	1	Putative homeodomain transcription factor 1	1.234	0.0274	4.78	3.64
OFD1	X	OFD1, centriole and centriolar satellite protein	1.230	0.00137	9.05	6.90
OSBPL3	7	Oxysterol binding protein-like 3	1.223	0.00921	4.80	3.73

Sorted by fold-change. F = females. M = males

^a^Benjamini-Hochberg false discovery rate

**Table 6:** Genes significantly upregulated in males (downregulated in females)

Gene symbol	Chr	Description	Fold-change (M/F)	FDR^a^	FPKM in F	FPKM in M
RPS4Y1	Y	Ribosomal protein S4, Y-linked 1	203.657	0	0.01	61.83
DDX3Y	Y	DEAD-box helicase 3, Y-linked	167.730	0	0.01	21.83
KDM5D	Y	Lysine demethylase 5D	160.898	0	0.00	6.73
USP9Y	Y	Ubiquitin specific peptidase 9, Y-linked	110.661	0	0.00	6.27
ZFY	Y	Zinc finger protein, Y-linked	83.865	0	0.00	5.66
UTY	Y	Ubiquitously transcribed tetratricopeptide repeat containing, Y-linked	83.286	0	0.00	7.99
TTTY15	Y	Testis-specific transcript, Y-linked 15 (non-protein coding)	78.249	0	0.00	4.42
EIF1AY	Y	Eukaryotic translation initiation factor 1A, Y-linked	62.683	0	0.01	6.11
LINC00278	Y	Long intergenic non-protein coding RNA 278	15.671	1.51E-127	0.02	5.44
TTTY14	Y	Testis-specific transcript, Y-linked 14 (non-protein coding)	13.548	4.73E-108	0.01	1.69
ANOS2P	Y	Anosmin 2, pseudogene	12.295	2.38E-102	0.01	1.28
PCDH11Y	Y	Protocadherin 11 Y-linked	12.042	7.38E-132	0.08	2.30
TXLNGY	Y	Taxilin gamma pseudogene, Y-linked	9.714	1.44E-75	0.00	1.37
ZFY-AS1	Y	ZFY antisense RNA 1	8.056	7.64E-66	0.02	1.97
PSMA6P1	Y	Proteasome subunit alpha 6 pseudogene 1	3.482	6.21E-27	0.40	2.02
ARMCX6	X	Armadillo repeat containing, X-linked 6	1.795	7.62E-06	1.78	3.95
MTRNR2L8	11	MT-RNR2-like 8	1.613	0.0201	2.90	4.88
ARMCX3	X	Armadillo repeat containing, X-linked 3	1.609	0.000255	6.70	11.93
HMGCS2	1	3-hydroxy-3-methylglutaryl-CoA synthase 2	1.603	0.035	1.99	4.27
FRG1JP	9	FSHD region gene 1 family member J, pseudogene	1.507	0.0209	1.36	2.27
HSPB1	7	Heat shock protein family B (small) member 1	1.486	0.0482	41.94	65.09
TLE2	19	Transducin-like enhancer of split 2	1.409	0.0495	2.61	3.67
ARHGEF9	X	Cdc42 guanine nucleotide exchange factor 9	1.344	0.0416	1.28	1.75

Sorted by fold-change. F = females. M = males

^a^Benjamini-Hochberg false discovery rate

As expected, most of the 58 differentially expressed genes (DEGs) are from the sex chromosomes (40/58; 69.0%). Twenty-five genes (43.1% of all DEGs) come from the X chromosome (18 protein-coding, 6 long non-coding, and 1 short non-coding), with 22 upregulated in females (Fig. 2b). Of all the X-linked genes upregulated in females, the majority, 13/22 (59.1%), were previously described to escape X chromosome inactivation (XCI). Of the remaining 9 genes, 4 underwent XCI and 5 had unknown XCI status [42, 46, 47]. Several (18/40; 31.0%) of the sex chromosome genes differentially expressed among the sexes in the late first trimester were identified to be sex different in term placenta tissue (Additional file 6), suggesting that they are important throughout gestation [20]. Differentially expressed autosome genes in the late first trimester identified here were unique and did not overlap with term placenta (Table 5 and 6, Additional file 6) nor with previous microarray studies comparing early first trimester terminations versus term placenta [20, 44].

We hypothesized that homologous X/Y gene pairs would be highly represented among differentially expressed sex-linked genes. First, we examined X-linked genes. Currently, there are 19 X-linked genes with known Y homologs outside of the pseudo-autosomal regions where the X and Y chromosomes may recombine, PAR1 and PAR2 [43]. Six of the 19 genes (31.6%) are upregulated in late first trimester female placentas and escape XCI in other tissue [42]. Furthermore, the corresponding Y homologs of these six X-linked genes are upregulated in late first trimester male placentas (Table 7).

**Table 7:** Chromosome X-linked genes with Y homologs

Chr X gene	XCI	▲ F	Chr X location	Gene descriptions	Chr Y gene	▲ M	Chr Y location
NLGN4X	E		p22.33	Neuroligin 4	NLGN4Y	A	q11.221
TBL1X	E		p22.3	Tranducin β-like 1	TBL1Y	A	p11.2
AMELX	–		p22.31-p22.1	Amelogenin	AMELY	A	p11.2
TMSB4X	–		q21.3-q22	Thymosin β4	TMSB4Y	A	q11.221
TXLNG	E	A	p22.2	Taxilin γ	TXLNGY	P	q11.222-q11.223
EIF1AX	E	P, A	p22.12	Eukaryotic translation initiation factor 1A	EIF1AY	P, A	q11.223
ZFX	E	P, A	p21.3	Zinc finger protein	ZFY	P, A	p11.2
USP9X	E		p11.4	Ubiquitin specific peptidase 9	USP9Y	P, A	q11.221
DDX3X	E	P, A	p11.3-p11.23	DEAD-box helicase 3	DDX3Y	P, A	q11.221
KDM6A	E	P, A	p11.2	Lysine demethylase	UTY	P, A	q11.221
TSPYL2	S		p11.2	Testis-specific protein Y-encoded (like 2)	TSPY1		p11.2
KDM5C	E	P, A	p11.22-p11.21	Lysine demethylase	KDM5D	P, A	q11.223
RPS4X	E	P, A	q13.1	Ribosomal protein S4	RPS4Y1	P, A	p11.2
TGIF2LX	–		q21.31	TGFβ-induced factor homeobox2-like	TGIF2LY		p11.2
PCDH11X	E		q21.3	Protocadherin 11	PCDH11Y	P, A	p11.2
XKRX	S		q22.1	XK, Kell blood group complex subunit-related	XKRY		q11.222
RBMX	S		q26.3	RNA binding motif	RBMY		q11.223
SOX3	S		q27.1	SRY-box 3/sex determining region Y	SRY	A	p11.2
HSFX1	–		q28	Heat shock transcriptional factor family	HSFY1		q11.222

Table of all X/Y homologs (excluding Y pseudogenes), sorted by location of X-linked genes. X chromosome inactivation (XCI) status is indicated as S (silenced/inactivated), E (escapes inactivation), or “–” if unstudied, as reviewed in Balaton et al. [42]. Sexually dimorphic expression is noted when genes are upregulated in first trimester placenta (“P”, this study) and adult postmortem tissue (“A”, Mele et al. 2015) [84]. The X genes listed are located within non-PAR regions. ▲ F = upregulated in females. ▲ M = upregulated in males

Next, we examined Y-linked genes. Only 16 Y-linked genes were expressed in the first trimester placenta and 15 were significantly differentially expressed (FDR < 0.05). Over half (8/15; 53.3%) of differentially expressed Y-linked genes are ancestral (*DDX3Y*, *EIF1AY*, *KDM5D*, *RPS4Y1*, *TXLNGY*, *USP9Y*, *UTY*, *ZFY*), all protein-coding except *TXLNGY.* These ancestral Y-linked genes are derived from the autosome-like proto-sex chromosomes of the last common therian ancestor of marsupial and placental mammals [39, 48, 49]. The X-linked homologs of 6 of these 8 genes were significantly upregulated in females, all except *TXLNG* (FDR = 0.468) and *USP9X* (FDR = 0.771). Males also expressed *PCDH11Y,* a human-specific Y-linked gene that arose from a relatively recent duplicative transposition from the X chromosome, 6 million years ago [49, 50]. *PCDH11X*, the X-linked homolog of *PCDH11Y*, was not differentially expressed between males and females in the first trimester placenta (FDR = 0.969). The remaining Y-linked genes were non-coding (Table 6).

**Fig. 3:**
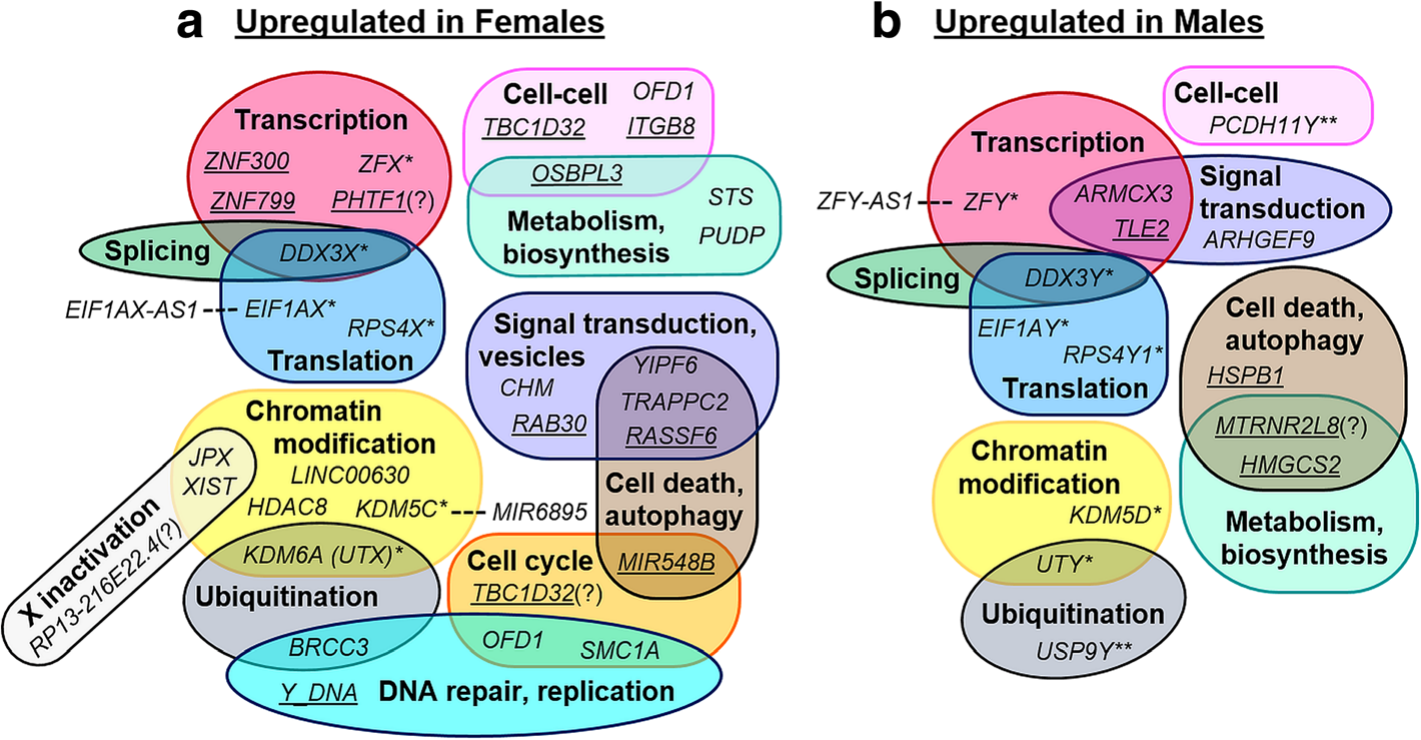
Functions of significantly differentially expressed genes in placenta. Venn diagrams of function categories for DEGs with known gene ontologies [39, 84, 85, 93, 97, 98, 100–106, 114–126]. Autosome genes are underlined. Sense/antisense pairs of DEGs are linked with dashed lines, with antisense genes outside the circles. *Homologous X/Y genes. **Genes with an X-linked homolog which is not differentially expressed. Functions predicted from sequence only are denoted with “(?)” next to the gene name. Genes without known or predicted function were omitted: *ANOS2P*, *ARMCX6*, *FRG1JP*, *IQCJ-SCHIP1-AS1*, *LINC00278*, *LINC00643*, *PSMA6P1*, *RP13-36G14.4*, *RPL23AP11*, *TTTY14*, *TTTY15*, and *TXLNGY*. **a** Genes upregulated in females. **b** Genes upregulated in males

For each of the 58 DEGs, gene ontologies were described and categorized into regulatory function groups when known (Fig. 3). Males and females both had upregulated genes involved in chromatin modification, transcription, splicing, translation, signal transduction, metabolic regulation, cell death and autophagy regulation, and ubiquitination. Some redundant functions were due to balanced expression of homologous X/Y genes. However, some of these categories also had an X-linked gene upregulated in males (*ARMCX3*) and differentially expressed autosome genes predominantly upregulated in females. Females also had more upregulated genes associated with signal transduction, DNA replication, cell cycle regulation, and regulation of various metabolites (e.g., hormone regulation by *STS*, dephosphorylation of pseudouridine 5′-phosphate by *PUDP*). Males had various upregulated genes from the Y chromosome without known function (Fig. 3b). Three sex-linked DEGs also had differentially expressed antisense genes upregulated in the same direction (Fig. 3), forming sense/antisense pairs of unknown, potentially regulatory function.

### Sex differences predominate over gestational age differences in CVS samples

We next sought to identify age-specific gene expression between early and late chorionic villus sampling (CVS) collection times. We performed subanalysis with week 11 samples (10.5-11.5 weeks at CVS) and week 13 samples (12.5–13.5 weeks at CVS) and found that samples separated by sex, but not gestational age (Additional files 7, 8, and 9). Therefore, we focused our subanalysis only on sex differences within subgroups. At week 11, there were 30 DEGs between male (*n* = 9) and female (*n* = 5) CVS samples (Additional file 10). Three new autosome genes (*STAT6*, *SLC4A1*, *LAMB3*) were significantly upregulated in males (FDR < 0.05). The remaining 17 sex-linked DEGs were consistent with the larger 39 sample analysis: 4 female-upregulated X-linked genes (*XIST*, *KDM6A*, *EIF1AX*, and *NUDT10*) and 13 male-upregulated Y-linked genes all had FDR < 0.05 in the 39 sample analysis (Additional files 4 and 10). At week 13, there were 20 DEGs between males (*n* = 4) and females (n = 4) (Additional file 10). Two new autosome genes were male-upregulated (*SERPINB7* and *PLTP*). Sex-linked DEGs were consistent with 39 sample results: female-upregulated X-linked genes (*XIST* and *KDM5C*), male-upregulated X-linked genes (*ARMCX3* and *ARMCX3*), and 14 Y-linked genes (all except *PSMA6P1*).

## Discussion

### Normative transcriptome and highest expressed genes consistent with a time of highly regulated rapid growth

The first trimester placenta is in a state of high transcriptional activity [7, 51]. We identified 14,250 genes expressed as part of the normative late first trimester placenta transcriptome. Chromosome 19 has the highest proportion of expression genes among all human chromosomes [52, 53], and this is reflected in the placental transcriptome as well. Chromosome Y was least expressed. However, the placenta transcriptome was enriched for ancestral Y-linked genes that have survived on the chromosome Y since its evolution from an autosome precursor, with 47.1% (8 of 17 total ancestral genes present in humans) expressed in late first trimester [39]. Expressed Y-linked genes were located in previously identified euchromatin regions, where DNA is less condensed and more accessible for transcription [54], consistent with the placental state of high transcriptional activity. Most (10/16; 62.5%) of expressed Y-linked genes have current or previous homology with the X chromosome. All eight protein-coding genes and one pseudogene (*TXLNGY*) have X homologs [54]. Another pseudogene (*ANOS2P)* is a “degraded homolog” of X-linked *ANOS1* gene, inactivated by a frameshift [55]. Notably, the testis-determining factor (*SRY* gene) is not expressed in the first trimester placenta, although it is expressed in male fetal cells as early as pre-implantation [56]. Together, this suggests that Y chromosome expression in the first trimester placenta may be supplementing the X chromosome in a pseudo-autosomal fashion, necessary for placental functions that may be distinct from fetal sex development.

The biotypes of expressed genes were skewed across FPKM distribution, with the highest expressed genes predominantly protein-coding genes. Short non-coding genes were expected to be underrepresented because the RNA extraction method is optimized for transcripts 200 nucleotides or above. Nevertheless, the two most expressed genes by FPKM were both short non-coding genes, *7SK* and *RN7SL2*. Human 7SK small nuclear RNA is abundant in animal tissue and critical for transcription elongation [57]. *RN7SL2* encodes 7SL RNA, an essential component of the signal recognition particle responsible for targeting proteins to the endoplasmic reticulum, a cell organelle important for protein secretion [58, 59]. Since placentation is a time of rapid cell proliferation and essential fetal-maternal communication, the high abundance of *7SK* and *RN7SL2* may be important to reduce growth restriction and facilitate protein secretion.

Gene enrichment analysis and upstream analysis of the top two quartiles of expressed genes showed a consistent pattern of expression in the placenta. Among the top 25% of genes, protein synthesis and cell growth pathways and functions were significantly enriched. Mitochondrial function and metabolic pathways were also significantly enriched, including the sirtuin signaling pathway involved in nutrient sensing and cellular metabolism [60]. Second quartile genes were enriched for hormone signaling pathways, and their binding partners (e.g., estradiol hormone) were found upstream of genes from the highest expressed quartile. Pregnancy maintenance is dependent on the interplay between hormone signaling and growth signals. The maintenance of the corpus luteum and its production of hormone during the first 8 weeks depends on trophectoderm-originated human chorionic gonadotropin (hCG) hormone [61], after which the placental production of progesterone and estrogen by the syncytiotrophoblast is sufficient to maintain pregnancy [62]. This is consistent with our findings demonstrating placental enrichment of hormone signaling canonical pathways with upstream regulation through estrogen. Other hormones such as insulin-like growth factors are also critical for regulating metabolism and fetal growth, especially in early pregnancy [63].

Finally, enriched pathways also show evidence of placental immune regulation activity. The JAK/STAT and STAT3 signaling pathway interacts with various cytokines, with mutations in pathway genes associated with both autoimmune and immunodeficient diseases [64]. Ceramide signaling in late first trimester placenta may be important for placental barrier integrity and maternal-fetal interactions, as ceramides are lipids important for skin’s barrier properties, yet excess ceramides are associated with pregnancy-induced hypertension and preeclampsia [65, 66].

### Several highly expressed protein-coding genes are placenta-enriched

Since placentation is a time of rapid growth, it was expected that essential and ubiquitously expressed genes such as ribosomal proteins would be among the highest expressed genes in the placenta. However, the highest expressed genes were not all housekeeping genes. Several had known and important functions in trophoblast growth and placental development. For example, *KISS1* (highest expressed protein-coding gene, third highest overall) is frequently found in overgrown tissue and tumors, yet seems to be protective against metastasis [67, 68]. In placentation, *KISS1* expression limits trophoblast invasion and migration by increasing cell adhesion to collagen, ensuring normal growth [69]. The second highest protein-coding gene was *glycoprotein hormone alpha polypeptide* (*CGA*), required for production of hCG, an important hormone produced by trophoblast cells days after conception and the biomarker for pregnancy tests [70–72]. To identify genes with placenta-specific or placenta-enriched expression, we searched for tissue expression profiles of the highest expressed protein-coding genes in the NCBI UniGene EST database for healthy tissue. Non-coding genes were omitted due to limited tissue expression data. Our in silico search showed that the highest expressed protein-coding genes in late first trimester placenta are not trivially expressed in all tissue types. Several genes have very limited tissue expression, placenta expression much higher than in other tissue, or both. Of the top 175 protein-coding genes in the first trimester, *X antigen family member 3* (*XAGE3*, also known as *PLAC6*) was the only gene known to be solely expressed in the placenta, out of 45 healthy body sites with possible transcript information for *XAGE3* on UniGene. *XAGE3* is part of a family of cancer/testis-associated antigens, although it is not expressed in either tumor or testis tissue, and its function is currently unknown [73]. BLAST of *XAGE3* showed high conservation among primates, poor conservation with non-primates (58% identity or less), and no significant hits in non-placental mammals, reinforcing the uniqueness of *XAGE3*.

Eleven histone-encoding genes highly expressed in first trimester placenta were not found in UniGene’s EST profile for placenta, have not been previously described in term placenta, and are also not among highly expressed genes in term placenta transcriptome studies [38, 44, 45], showing that the first trimester placenta transcriptome is different from term placenta. Histones are DNA-binding proteins responsible for packaging DNA into nucleosomes, and differences in histones and histone modifications are associated with differences in DNA accessibility for transcription. The high expression of these histone-encoding genes in the first trimester placenta, but not later in gestation, suggests that the first trimester chromatin structure is unique and functions to maintain a high transcriptionally active state. Eight of these histone genes (*HIST1H1B*, *HIST1H1E*, *HIST1H3B*, *HIST1H3C*, *HIST1H3F*, *HIST1H4B*, *HIST1H4D*, and *HIST1H4F*) had very limited tissue expression in non-placental tissue, primarily appearing in embryonic, vascular, skin, and connective tissue. Although these genes are not significantly sex-different in placenta, all eight are significantly upregulated in response to testosterone in mouse embryonic stem cells, suggesting an additional mechanism by which the prenatal hormone environment may affect placental and fetal gene expression [74]. A ninth gene, *HIST1H2BI*, had no significant UniGene-documented expression in any normal tissue, showing EST count of 1/1092688 (effectively TPM = 0) in fetal brain, and no EST counts in any other normal or abnormal tissue. Its abundance in first trimester placenta (mean FPKM = 187, TPM = 564) suggests that its tissue and developmental stage expression is heavily regulated. Together, these results suggest that first trimester placenta RNA transcription is tightly controlled and possibly contains a novel histone profile that is different from both term placenta and adult tissue.

### Sex differences exist in late first trimester placenta gene expression

Out of 58 DEGs in the late first trimester placenta, over a third (22/58; 37.9%) were X-linked genes upregulated in female samples. X chromosome inactivation in females is initiated during early embryonic development, when one X chromosome is randomly silenced to achieve dosage compensation between XX females and XY males. About 12% of X-linked genes always escape X inactivation and about 8% additional X-linked genes variably escape X inactivation in humans [75]. In our first trimester placenta results, over half (13/22; 59.1%) of X-linked genes upregulated in females are reported to escape XCI based on previous studies [42]. This overrepresentation of genes known to escape X inactivation in female first trimester placenta may be the result of a necessary double dose effect, similar to a double dose effect described with *DAX* [76]. This suggests that the increased expression from genes that escape XCI may be due to expression from both chromosomes.

XCI studies in early and term human placentas have been controversial. In earlier studies, XCI in the first trimester human placenta was reported either random in some studies or preferentially paternal in others [77–80]. In term placentas, relatively large patches of placental cells show either maternal or paternal inactivated X and the chorionic villous trees are clonally derived from only one or a few precursor cells [81, 82]. Our first trimester placenta samples consist of individual chorionic villous trees and thus might represent clonality. However, since each chorionic villus is composed of heterogenous cell populations (including trophoblast cells, mesenchymal cells, fetal endovascular cells) and different cell types have different gene expression patterns as already shown in term placenta [20, 83], the clonality of allelic specific expression in individual villi of the first trimester placenta remains to be determined.

Furthermore, the sexual dimorphic expression patterns of X-linked genes in the first trimester placentas from this study resemble what was identified by a recent transcriptome analysis in adult human tissues [84]. Sexually dimorphic expression patterns that are highly conserved from early placenta into adulthood may indicate important systemic sex differences. Among the 22 genes upregulated in females in first trimester placenta, 14 (63.6%) were also upregulated in females across multiple non-placental adult human tissues [84]. Almost all (11/14; 78.6%) were identified to escape XCI [42], with the exception of *XIST* which is X-inactivated, and *EIF1AX-AS1* and *RP13-216E22.4* whose XCI statuses are unknown. This suggests that the upregulation of these X-linked genes in females may be due to expression from both chromosomes, beginning in the first trimester and continuing into adulthood. These genes may be critical markers of sex differences in fetal growth and development that transcends into adulthood determining sex differences in adult diseases, known as fetal origins of adult diseases.

However, not all sexually dimorphic genes matched sex differences in adult tissues [84]. There were eight X-linked genes that were upregulated in females only in the late first trimester placentas: *BRCC3*, *CHM*, *HDAC8*, *LINC00630*, *MIR6895*, *OFD1*, *RP13-36G14.4*, and *YIPF6.* These genes play important roles in transcriptional regulation, DNA damage response, and vesicle-mediated transport, suggesting biological processes that may be sex-biased and critical for early placenta development. *LINC00630* promotes cell proliferation by promoting the protein stability of histone deacetylate HDAC1 [85]. Non-coding gene *MIR6895* is antisense to histone demethylase-encoding *KDM5C*, and thus may also regulate placental chromatin status*.* Among males, 13 Y-linked genes were consistent with that in the adult tissues, but pseudogenes *ANOS2P* and *PSMA6P1* exhibited male-upregulation only in the first trimester placenta. Similar to other pseudogenes, they may act as miRNA decoys which may be important in early development [86, 87]. Three X-linked genes *ARMCX3* (armadillo repeat containing, X-linked 3), *ARMCX6* (armadillo repeat containing, X-linked 6), and *ARHGEF9* (Cdc42 guanine nucleotide exchange factor 9) were also upregulated in male placenta, but not adult male tissues [84]. The three upregulated X-linked genes in males typically undergo X inactivation in other tissue, indicating that one active allele is likely sufficient for their function [42]. It remains to be determined whether the increased gene levels of these three X-linked genes in males is due to upstream Y-linked genes or if the second X chromosome downregulates these genes in females. Though there is limited research, male-upregulated expression of X-linked genes has been reported previously [84, 88]. Alex3 (encoded by *ARMCX3*) regulates migration and invasion in tumor cells, functions which are also critical for placentation and subsequent fetal outcomes, including fetal growth [89].

Autosome genes comprised almost a third of DEGs (18/58; 31.0%) in late first trimester placenta. In silico upstream analysis of the autosome DEGs did not yield any regulators on the sex chromosomes nor hormonal regulators that may explain the differences in expression between males and females, though this may be due to limitations of currently available data. Although many of the sex chromosome DEGs have been found in previous studies of sex differences, none of the autosome DEGs are previously identified as sex different in term placenta [20]. One autosome gene (*RASSF6* on chromosome 4) was significantly upregulated in females in both late first trimester placenta and adult breast tissue [84]. Since both placenta and adult breast tissue are responsive to sex hormones, this overlap in *RASSF6* makes biological sense. Overall, sex differences in autosome gene expression exist, but appear to be more subject to age- and tissue-specific variability than sex chromosome DEGs.

### Distribution of sexual dimorphisms along parallel pathways (X/Y homologs)

Over a fourth (6/22; 27.3%) of X-linked genes upregulated in females have functional homologs on the Y chromosome, including *EIF1AX*, *DDX3X*, *KDM5C*, *KDM6A*, *RPS4X*, and *ZFX*. However, not all X-linked genes with Y homologs are sexually dimorphic. In fact, when examining significantly different expressed Y-linked genes in male, three Y-linked genes (*PCDH11Y*, *TXLNGY*, and *USP9Y*) had functional X-homologs that displayed balanced expression between the sexes. *USP9Y* was once thought to be essential for spermatogenesis, but men with complete deletions of *USP9Y* can be fertile [90, 91]. Polymorphisms in the *USP9Y* gene affect serum lipid profiles and coronary heart disease risk, showing that this Y-linked gene has a non-gametogenesis function [92]. The expression of three Y-linked genes in male placenta without balancing upregulation of X-linked homologs in female placentas suggests that these genes may have additional important functions for placental development that may have long-term health implications for sexually dimorphic diseases.

With pathways analysis of the 58 DEGs, we found few predicted whole pathway differences between males and females (not shown). This may be due to autosome-like expression of X/Y gene pairs such as *EIF1AX/EIF1AY* and *RPS4X/RPS4Y1*. Although the individual protein-coding genes show sexually dimorphic expression, the canonical pathways are not differentially “activated” or “inactivated,” but instead function in parallel in the same direction. We saw this dimorphic-but-parallel expression in chromatin modification, transcription, splicing, and translation pathways.

### Functional significance of sexually dimorphic genes

Sexually dimorphic genes are likely functionally relevant for placenta biology. Females had a greater number of upregulated genes encoding DNA binding proteins, including genes associated with chromatin modification (including X inactivation) and genes encoding components of centrioles and cohesin complexes (*OFD1* and *SMC1A*, respectively) important for segregation of replicated DNA during cell division [93, 94]. Metabolism-associated upregulated genes in males were primarily involved in response to nutrient deficit. *MTRNR2L8* (1.61-fold upregulated in males) encodes Humanin-like 8, a small peptide homologous and sometimes identical (due to a polymorphic site) to the mitochondrially encoded Humanin peptide which promotes cell survival in ATP-deficient environments [95, 96]. Humanin promotes insulin sensitivity which may contribute to increased fetal growth seen in males [97]. *HMGCS2* (1.60-fold upregulated in males) encodes an enzyme that promotes autophagy by catalyzing the first step in ketogenesis, a pathway that derives energy from lipids when carbohydrates are depleted [98]. This is consistent with previous pregnancy studies that find greater risk for nutrient deficit in males [99]. In contrast, metabolism-associated genes upregulated in females are involved in post-transcriptional modification of RNA (*PUDP*) and hormone biosynthesis (*STS*) [100, 101]. Cell adhesion, ciliogenesis, and cell-cell communication genes (*OFD1*, *OSBL3*, *PCDH11Y*, *TBC1D32*) were also differentially expressed, suggesting sex differences in how placenta cells interact with their environment [102–105]. *ITGB8* (encodes integrin-β8) promotes tumor angiogenesis and invasiveness in glioblastoma [105], functions necessary for normal first trimester development when placental cells invade maternal tissue and access maternal blood.

Differentially expressed transcription factors in first trimester may explain sex differences in later pregnancy complications. For example, *ZNF300* (1.58-fold upregulated in females) encodes a transcription factor protein that shares DNA binding sites with Early growth response 1 (Egr1), a zinc finger transcription factor that is elevated in placentas of pregnancies that later develop preeclampsia, which is more common in pregnancies with a male fetus [15, 106, 107]. Increased expression of *ZNF300* in females may function as a competitor of *EGR1* binding sites, leading to reduced rates of preeclampsia. Further studies of *ZNF300* and other sex different genes are needed to understand their biological roles in placental development.

### Y-linked and chromatin modification genes are consistently sex different

Gestational age-specific sex differences exist, even between 11 and 13 weeks gestation. We found that a subset of Y-linked genes are consistently expressed in late first trimester placenta regardless of gestational age and into adulthood, whereas X-linked genes and autosome genes were more variable. However, X-linked genes that affect chromatin modification (*XIST*, *KDM6A*, *KDM5C*) are conserved throughout gestation and into adulthood [84].

### Sex differences of fetal weight at birth

In our cohort, male infants had higher birth weights compared to female infants, consistent with known sex differences at birth [108, 109]. Sex differences in embryonic and fetal growth rates have been long recognized [110], but the underlying mechanisms remain poorly understood. Recent studies show associations between placental biomarkers and fetal growth [111–113]. Since sex differences in gene expression may be implicated in placental function, altering growth and development which can translate into sexual dimorphism of disease in adulthood, further studies are necessary to better understand the functional roles of these genes in early pregnancy.

### Limitations

All subjects in this study were Caucasian. Caucasians make up the largest demographic for chorionic villus sampling tissue that is available through our prenatal biorepository. It remains to be seen if sex differences in placenta transcripts vary among different races. Additionally, the normative placenta transcriptome described for the late first trimester here may be underrepresenting short RNAs. We found that 6.7% of the overall transcribed genes were short non-coding RNAs. The RNA extraction method used prior to RNA-seq is optimized for RNA of lengths > 200 nt, which loses 5S rRNA, tRNAs, mature miRNAs, and several other (though not all) short RNAs. Furthermore, although we compared differential expression from the late first trimester to previously published data looking at term placenta, results need to be interpreted with caution since term placenta studies were performed using microarrays with smaller sample sizes and not RNA-seq.

## Conclusions

This is the largest study to date that identifies the late first trimester human placenta transcriptome, highlighting similarities and differences among the sexes. This is the earliest time point that ongoing human pregnancies that result in a live birth can be studied. We identified a group of 11 histone-encoding genes which were highly expressed in first trimester placenta, but have not been previously reported in term placenta, suggesting they may have temporally specific functions important for early pregnancy regardless of sex. When examining our cohort of patients for sex differences, we found 58 significantly differentially expressed genes with an overrepresentation of genes known to escape X inactivation. Many of the sexually dimorphic genes fell into a group of X/Y gene pairs, suggesting that dosage compensation plays a role in sex differences. Gene ontology analysis of differentially expressed genes suggested molecular signaling differences that may affect pregnancy outcomes, but further study of these genes in placenta models is needed. This is the first step into the characterization of normal placental function that can lead to the development of diagnostic tests for the normal and dysfunctional placenta, as well as identification of sex differences that start in utero and may translate into sex differences of adult diseases. This study can be used to develop a sex-specific placenta atlas which will be unique to humans at this early time point in placental development.

## Additional files

Additional file 1: RNA-sequencing figures for 39 CVS analysis. **a** Principal components analysis plot of the 39 CVS samples shows male and female clusters. Blue: male samples, red: female samples. **b** Volcano plot of RNA-seq results before FPKM cutoff. **c** MA plot of RNA-seq results before FPKM cutoff. (DOC 1937 kb)

Additional file 2: Table S1. Expressed genes in late first trimester human placenta, sorted by FPKM. Ensembl IDs, gene symbols, biotypes, FPKM values, baseMean values, gene descriptions, and statistics from DESeq2 are given. (XLS 25631 kb)

Additional file 3: Table S2. Top 40 upstream regulators for the top 25% of expressed genes. (DOC 84 kb)

Additional file 4: Table S3. Sex different genes in late first trimester placenta. DESeq2 results for FDR < 0.05 genes before FPKM cutoff. (XLS 243 kb)

Additional file 5: Table S4. Cell marker expression in 39 CVS samples. Cell types in CVS tissue appear to be similar in male and female samples. No cell marker is significantly sex different. (DOC 48 kb)

Additional file 6: Table S5. Sex differences in CVS compared to previous term placenta study. Overlap of DEGs in the current study and DEGs in epithelium and endothelium in term placenta. (DOC 60 kb)

Additional file 7: Principal components analysis for week 11 vs week 13 subgroups. Early and late CVS collection time does not separate the samples. Green: week 11 samples. Purple: week 13 samples. (TIFF 610 kb)

Additional file 8: Week 11 DESeq2 subanalysis for sex differences. **a** Principal components analysis for week 11 subgroup shows male and female clusters. **b** Volcano plot before FPKM cutoff. **c** MA plot before FPKM cutoff. (DOC 1071 kb)

Additional file 9: Week 13 DESeq2 subanalysis for sex differences. **a** Principal components analysis for week 13 subgroup shows male and female clusters. **b** Volcano plot before FPKM cutoff. **c** MA plot before FPKM cutoff. (DOC 863 kb)

Additional file 10: Table S6 and Table S7. Sexually dimorphic genes in human placenta at weeks 11 and 13. Spreadsheet of significantly different genes for gestational age subanalysis (female vs male, FDR < 0.05). Table S6: week 11. Table S7: week 13. (XLS 70 kb)

## Abbreviations

BMI: Body mass index
CRL: Crown-rump length
CVS: Chorionic villus sampling
DEG: Differentially expressed gene
EST: Expressed sequence tag
FDR: False discovery rate
FPKM: Fragments per kilobase per million mapped fragments
GEO: Gene Expression Omnibus
hCG: Human chorionic gonadotropin
IPA: Ingenuity Pathways Analysis
IUGR: Intrauterine growth restrictions
NCBI: National Center for Biotechnology Information
RIN: RNA integrity number
TPM: Transcripts per million
XCI: X chromosome inactivation
