# Supplementary material for: Sex differences in the late first trimester human placenta transcriptome
Source: Biol Sex Differ. 2018 Jan 15;9:4. doi: 10.1186/s13293-018-0165-y (PMC5769539; doi:10.1186/s13293-018-0165-y)

**Additional file 1.** Analysis of the 39 CVS samples used for female vs male RNA-sequencing and DESeq2.

**(A)** **Principal components analysis shows male and female clusters.** Principal components analysis plot of the 39 CVS samples used for RNA-seq shows clustering by cytogenetic sex. Blue: male samples, red: female samples.


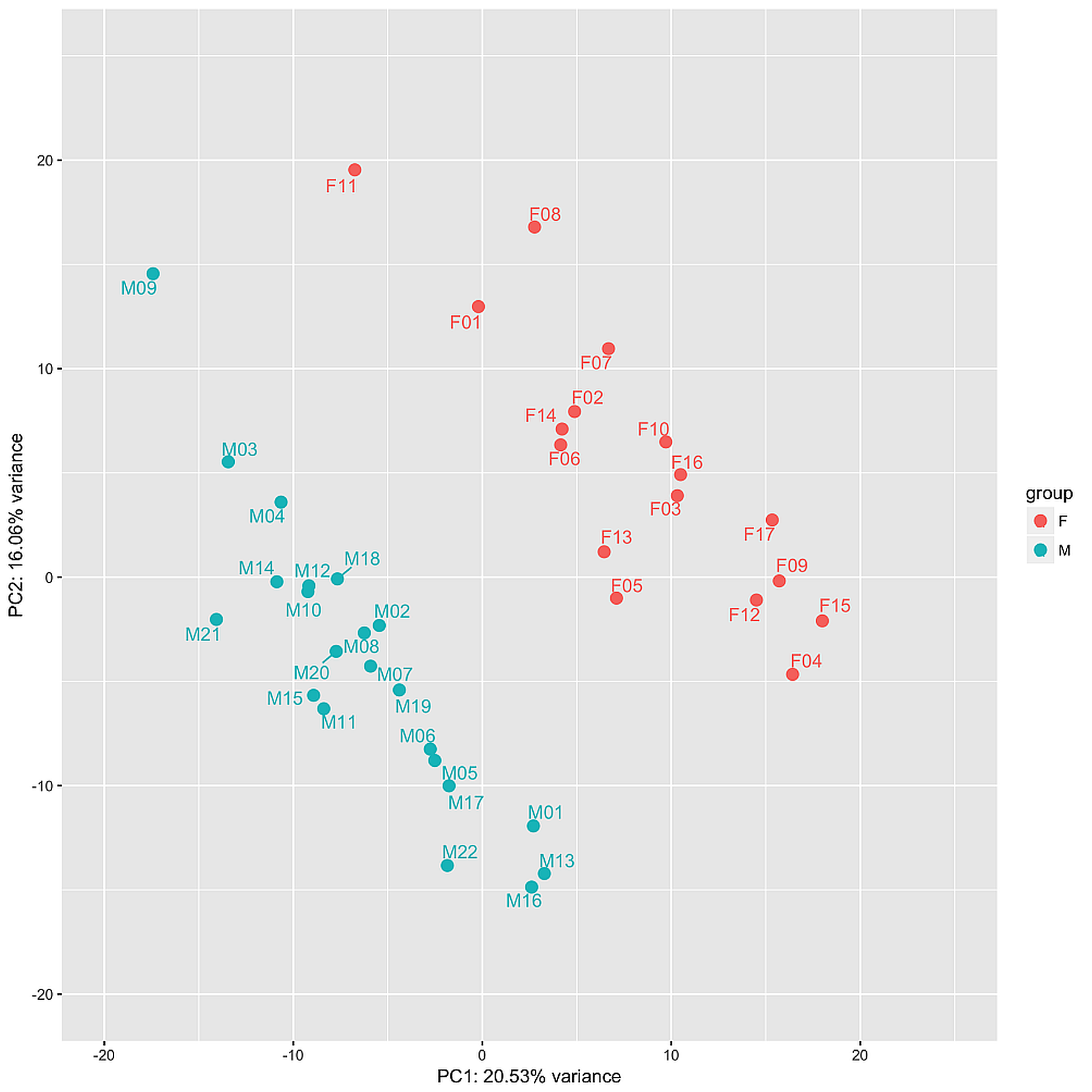


**(B)** **Volcano plot of RNA-seq results before FPKM cutoff.** DESeq2 results for sex differences in 39 CVS samples. Volcano plot for all genes detected by RNA-seq, without an FPKM cutoff for expression. Red points: genes significantly different between sexes (Benjamini-Hochberg procedure to control for false discovery rate, FDR<0.05). Yellow points: genes with 2-fold change or greater, |LogFC|>1 where FC = female/male fold-change. No genes fit yellow category. Blue points: genes which are both significantly different (FDR<0.05) and have a 2-fold change or greater difference in expression between males and females. Black points: other genes.


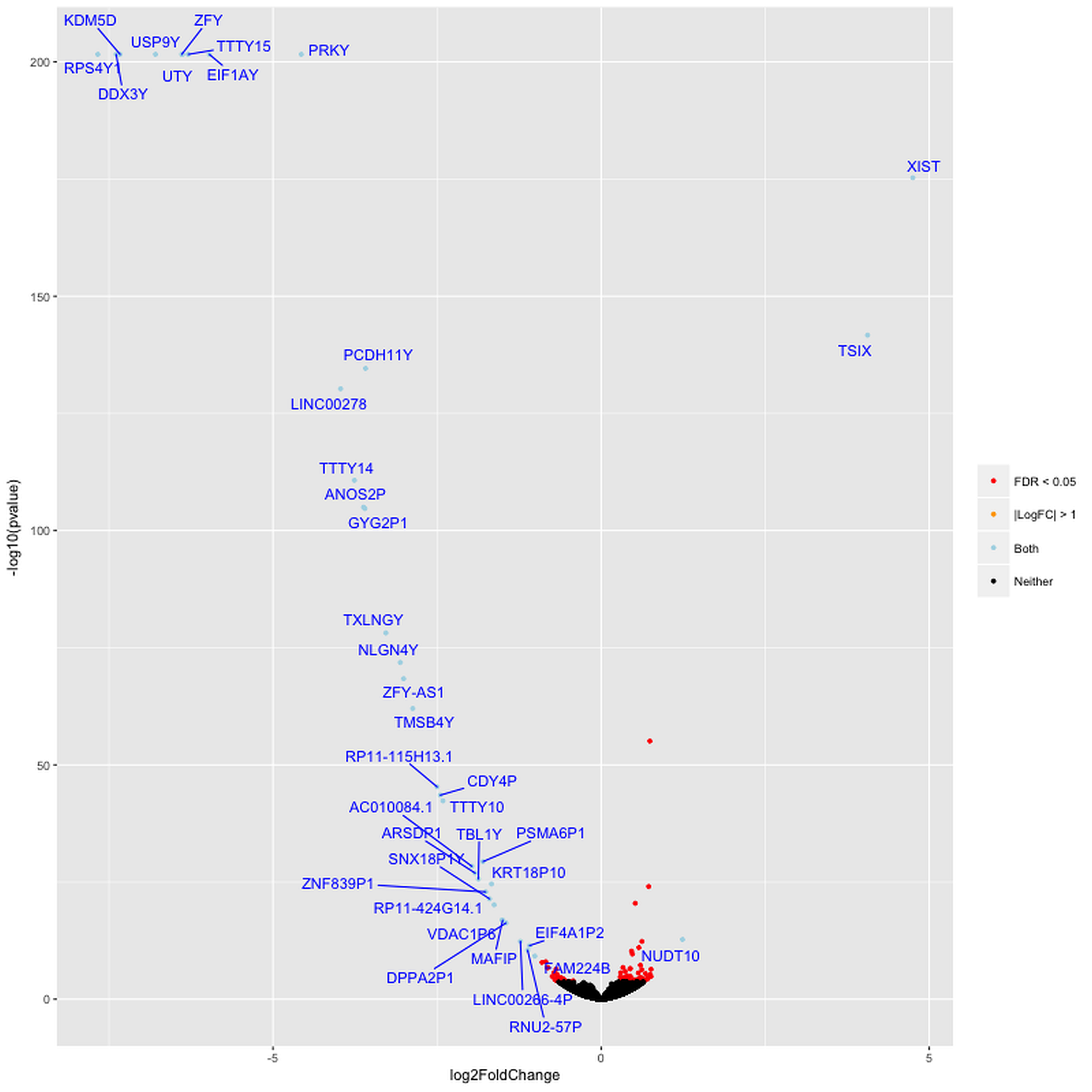


**(C) MA plot of RNA-seq results before FPKM cutoff.** DESeq2 results for sex differences in 39 CVS samples, before an FPKM cutoff is applied. MA plot shows log2 of fold-changes (female/male) versus baseMean expression values (gene counts normalized by sequencing depth). Red points: genes significantly different between sexes (Benjamini-Hochberg procedure to control for false discovery rate, FDR<0.05). Black points: other genes.


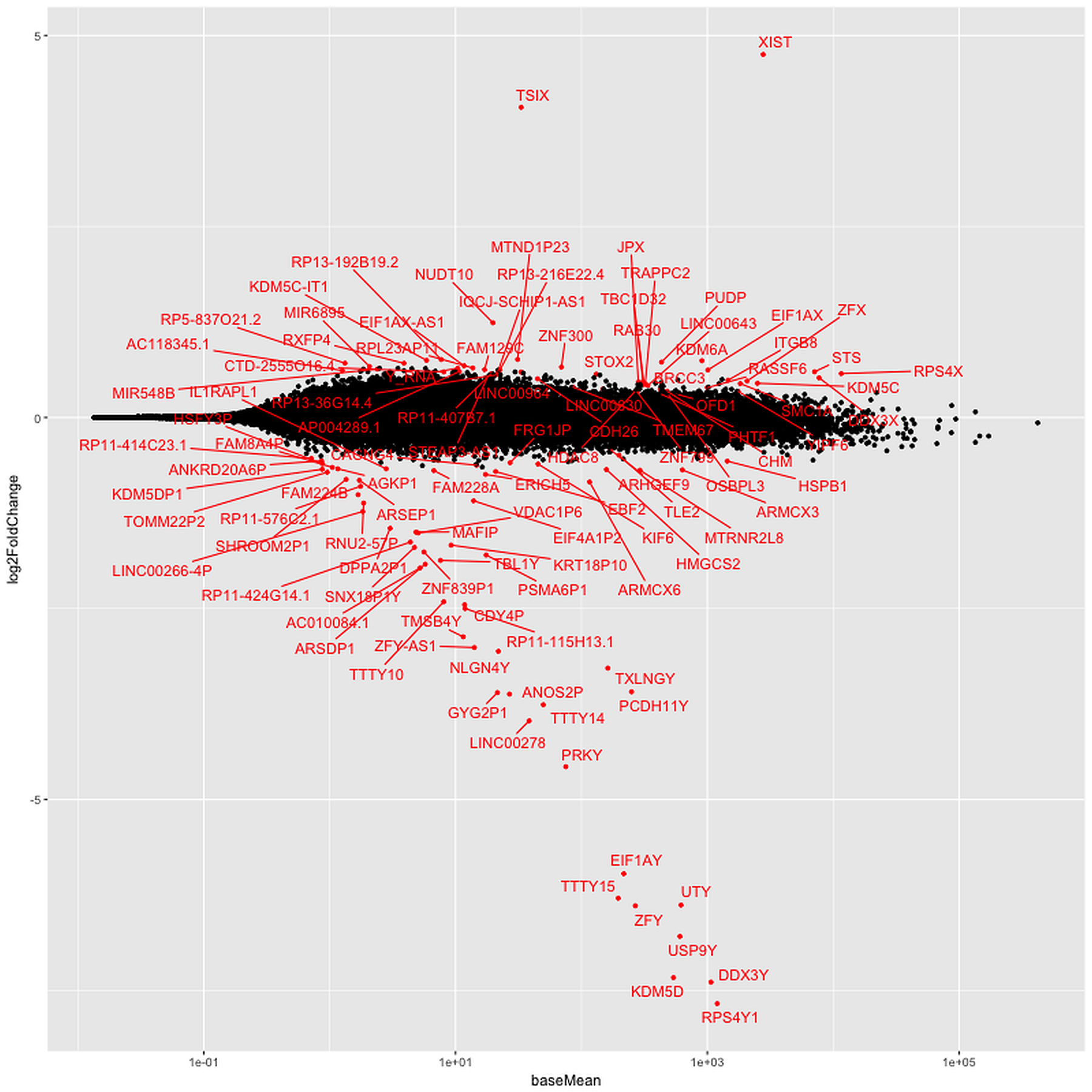

Supplement: Supplementary file 1 — RNA-sequencing figures for 39 CVS analysis. a Principal components analysis plot of the 39 CVS samples shows male and female clusters. Blue: male samples, red: female samples. b Volcano plot of RNA-seq results before FPKM cutoff. c MA plot of RNA-seq results before FPKM cutoff. (DOC 1937 kb) [file 13293_2018_165_MOESM1_ESM.doc]
