# Supplementary material for: Sex differences in the late first trimester human placenta transcriptome
Source: Biol Sex Differ. 2018 Jan 15;9:4. doi: 10.1186/s13293-018-0165-y (PMC5769539; doi:10.1186/s13293-018-0165-y)
Supplement: Supplementary file 3 — Top 40 upstream regulators for the top 25% of expressed genes. (DOC 84 kb) [file 13293_2018_165_MOESM3_ESM.doc]

**Table S2. Top 40 upstream regulators for the top 25% of expressed genes.**

| **#** | **Upstream Regulator** | **Description** | **Molecule Type** | **Overlap**  **P-value^a^** | **% Genes Regulated** |
| --- | --- | --- | --- | --- | --- |
| 1 | MYC | MYC proto-oncogene, bHLH transcription factor | transcription regulator | 1.70E-70 | 11.6% |
| 2 | TP53 | tumor protein p53 | transcription regulator | 1.63E-65 | 14.3% |
| 3 | MYCN | MYCN proto-oncogene, bHLH transcription factor | transcription regulator | 3.17E-63 | 4.8% |
| 4 | CD437 | retinoic acid receptor (RAR)γ-selective agonist | chemical drug | 1.69E-62 | 4.0% |
| 5 | sirolimus | rapamycin | chemical drug | 2.79E-59 | 6.7% |
| 6 | HNF4A | hepatocyte nuclear factor 4 alpha | transcription regulator | 2.10E-58 | 17.1% |
| 7 | ST1926 | adarotene;  synthetic, phenolic hydroxyl retinoid | chemical drug | 2.21E-58 | 3.3% |
| 8 | 5-fluorouracil | Pyrimidine analog | chemical drug | 4.37E-55 | 4.3% |
| 9 | RICTOR | RPTOR independent companion of MTOR complex 2 | other | 4.58E-46 | 4.0% |
| 10 | beta-estradiol | Steroid sex hormone | chemical - endogenous mammalian | 5.14E-46 | 14.5% |
| 11 | HRAS | HRas proto-oncogene, GTPase | enzyme | 2.99E-42 | 6.5% |
| 12 | ERBB2 | erb-b2 receptor tyrosine kinase 2 | kinase | 1.21E-40 | 7.6% |
| 13 | TGFB1 | transforming growth factor beta 1 | growth factor | 1.55E-40 | 13.9% |
| 14 | ESR1 | estrogen receptor 1 | ligand-dependent nuclear receptor | 1.51E-39 | 11.0% |
| 15 | KRAS | KRAS proto-oncogene, GTPase | enzyme | 3.32E-34 | 5.4% |
| 16 | APP | amyloid beta precursor protein | other | 1.30E-30 | 7.8% |
| 17 | CD3 | transmembrane receptor complex | complex | 4.66E-30 | 6.1% |
| 18 | D-glucose | Monosaccharide | chemical - endogenous mammalian | 5.68E-30 | 5.6% |
| 19 | dexamethasone | synthetic adrenal corticosteroid | chemical drug | 3.82E-29 | 12.6% |
| 20 | SYVN1 | synoviolin 1 | transporter | 5.93E-28 | 2.2% |
| 21 | dihydrotestosterone | biologically active metabolite of testosterone | chemical - endogenous mammalian | 7.33E-28 | 5.3% |
| 22 | EGFR | epidermal growth factor receptor | kinase | 1.27E-27 | 4.3% |
| 23 | 1,2-dithiol-3-thione | 3H-1,2-dithiole-3-thione | chemical reagent | 1.96E-27 | 2.8% |
| 24 | XBP1 | X-box binding protein 1 | transcription regulator | 8.12E-27 | 2.8% |
| 25 | PGR | progesterone receptor | ligand-dependent nuclear receptor | 3.24E-26 | 3.3% |
| 26 | miR-124-3p (and other miRNAs w/seed AAGGCAC) | hsa-miR-124-3p | mature microrna | 4.26E-26 | 3.0% |
| 27 | EGF | epidermal growth factor | growth factor | 8.02E-26 | 5.0% |
| 28 | hydrogen peroxide | H_2_O_2_ | chemical - endogenous mammalian | 6.44E-25 | 4.8% |
| 29 | PTEN | phosphatase and tensin homolog | phosphatase | 8.67E-25 | 4.6% |
| 30 | MAPT | microtubule associated protein tau | other | 3.09E-24 | 3.1% |
| 31 | tretinoin | retinoic acid; vitamin A acid | chemical - endogenous mammalian | 5.13E-24 | 11.2% |
| 32 | FN1 | fibronectin 1 | enzyme | 6.10E-24 | 2.7% |
| 33 | FOS | Fos proto-oncogene, AP-1 transcription factor subunit | transcription regulator | 6.28E-24 | 5.1% |
| 34 | butyric acid | saturated short-chain fatty acid | chemical - endogenous mammalian | 7.19E-24 | 4.4% |
| 35 | FSH | follicle-stimulating hormone (CGA, FSHB) | complex | 2.22E-23 | 4.1% |
| 36 | INSR | insulin receptor | kinase | 4.02E-23 | 4.0% |
| 37 | mibolerone | synthetic androgen;  7alpha-17alpha-dimethyl-19-nortestosterone | chemical drug | 4.74E-23 | 2.0% |
| 38 | NFE2L2 | nuclear factor, erythroid 2 like 2 | transcription regulator | 8.00E-23 | 4.2% |
| 39 | PSEN1 | presenilin 1 | peptidase | 9.84E-23 | 4.0% |
| 40 | FLT1 | fms related tyrosine kinase 1 | kinase | 2.26E-22 | 1.4% |

^a^P-value: Fisher’s Exact Test. After RNA-sequencing of 39 CVS samples, the top 25% of expressed genes (3563 genes) was input into IPA, with 3493 genes accepted for analysis.
