# Supplementary material for: Sex differences in the late first trimester human placenta transcriptome
Source: Biol Sex Differ. 2018 Jan 15;9:4. doi: 10.1186/s13293-018-0165-y (PMC5769539; doi:10.1186/s13293-018-0165-y)
Supplement: Supplementary file 5 — Cell marker expression in 39 CVS samples. Cell types in CVS tissue appear to be similar in male and female samples. No cell marker is significantly sex different. (DOC 48 kb) [file 13293_2018_165_MOESM5_ESM.doc]

**Table S4. Cell marker expression in 39 CVS samples.**

| **Ensembl Gene ID** | **Marker For** | **Gene Symbol** | **Chr** | **Description** | **FDR^a^** | **FPKM** in F | **FPKM**  in M |
| --- | --- | --- | --- | --- | --- | --- | --- |
| ENSG00000135480 | Epithelial; TB | **KRT7** | 12 | keratin 7 | 0.480 | 120.3046 | 137.1856 |
| ENSG00000165556 | Trophectoderm; early 1^st^ trimester CTB | **CDX2** | 13 | caudal type homeobox 2 | 0.894 | 0.0614 | 0.0457 |
| ENSG00000163508 | Trophectoderm; early 1^st^ trimester CTB | **EOMES** | 3 | eomesodermin | 0.88 | 0.0146 | 0.0145 |
| ENSG00000197905 | CTB | **TEAD4** | 12 | TEA domain transcription factor 4 | 0.839 | 10.3369 | 10.5036 |
| ENSG00000073282 | CTB | **TP63** | 3 | tumor protein p63 | 0.293 | 14.0153 | 10.8696 |
| ENSG00000010278 | EVT | **CD9** | 12 | CD9 molecule | 0.947 | 5.4304 | 5.3548 |
| ENSG00000204632 | EVT | **HLA-G** | 6 | major histocompatibility complex, class I, G | 0.886 | 8.6067 | 14.1601 |
| ENSG00000087245 | EVT | **MMP2** | 16 | matrix metallopeptidase 2 | 0.489 | 34.0737 | 51.9172 |
| ENSG00000100985 | EVT | **MMP9** | 20 | matrix metallopeptidase 9 | 0.947 | 3.0406 | 2.3373 |
| ENSG00000104827 | STB | **CGB3** | 19 | chorionic gonadotropin beta subunit 3 | 0.605 | 463.7882 | 884.3069 |
| ENSG00000189052 | STB | **CGB5** | 19 | chorionic gonadotropin beta subunit 5 | 0.472 | 655.3999 | 1353.0732 |
| ENSG00000213030 | STB | **CGB8** | 19 | chorionic gonadotropin beta subunit 8 | 0.492 | 743.9698 | 1397.0911 |
| ENSG00000146648 | STB | **EGFR** | 7 | epidermal growth factor receptor | 0.782 | 102.6326 | 93.9979 |
| ENSG00000243137 | STB | **PSG4** | 19 | pregnancy specific beta-1-glycoprotein 4 | 0.902 | 264.2003 | 345.6462 |
| ENSG00000164692 | Fibroblast | **COL1A2** | 7 | collagen type I alpha 2 chain | 0.700 | 89.8895 | 101.7816 |
| ENSG00000138685 | Fibroblast | **FGF2** | 4 | fibroblast growth factor 2 | 0.909 | 1.0802 | 1.0083 |
| ENSG00000026025 | Mesenchymal | **VIM** | 10 | vimentin | 0.848 | 43.5063 | 45.3028 |

^a^Benjamini-Hochberg False Discovery Rate. Cell types in CVS tissue appear to be similar in male (M) and female (F) samples. No cell marker is significantly sex different.

TB = trophoblast (general cell type)

CTB = cytotrophoblast (progenitor cell type for EVTs and STBs)

EVT = extravillous trophoblast

STB = syncytiotrophoblast

**Differentiation:** trophectoderm 🡪 cytotrophoblast 🡪 extravillous trophoblast or syncytiotrophoblast
