# Supplementary material for: Sex differences in the late first trimester human placenta transcriptome
Source: Biol Sex Differ. 2018 Jan 15;9:4. doi: 10.1186/s13293-018-0165-y (PMC5769539; doi:10.1186/s13293-018-0165-y)
Supplement: Supplementary file 6 — Sex differences in CVS compared to previous term placenta study. Overlap of DEGs in the current study and DEGs in epithelium and endothelium in term placenta. (DOC 60 kb) [file 13293_2018_165_MOESM6_ESM.doc]

**Additional file 6.** Overlap of DEGs in the current study and DEGs in epithelium and endothelium in term placenta (M = male, F = female).

| **Sex differences in CVS Tissue (RNA-sequencing)**  **This study** | | | | **Sex differences in cultured term placenta tissue (RNA microarray)**  **Cvitic *et al*. 2013 study** | | | | | |
| --- | --- | --- | --- | --- | --- | --- | --- | --- | --- |
| **Ensembl Gene ID** | **Gene Symbol** | **Chr** | **CVS direction** | **Term epithelium (STC and CT) direction** | **Term endothelium (AEC and VEC) direction** | **Term SCT direction** | **Term CT direction** | **Term AEC direction** | **Term VEC direction** |
| ENSG00000198960 | ARMCX6 | X | M | M |  |  |  |  |  |
| ENSG00000188419 | CHM | X | F |  | F |  |  | F | F |
| ENSG00000215301 | DDX3X | X | F |  |  |  |  |  | F |
| ENSG00000067048 | DDX3Y | Y | M | M | M | M | M | M | M |
| ENSG00000173674 | EIF1AX | X | F |  | F | F |  | F | F |
| ENSG00000198692 | EIF1AY | Y | M | M | M | M | M | M | M |
| ENSG00000126012 | KDM5C | X | F | F | F |  | F | F | F |
| ENSG00000012817 | KDM5D | Y | M | M | M | M | M | M | M |
| ENSG00000147050 | KDM6A | X | F | F | F |  | F | F |  |
| ENSG00000099715 | PCDH11Y | Y | M | M |  |  | M |  |  |
| ENSG00000198034 | RPS4X | X | F |  |  |  |  | F |  |
| ENSG00000129824 | RPS4Y1 | Y | M | M | M | M | M | M | M |
| ENSG00000072501 | SMC1A | X | F | F | F |  | F | F | F |
| ENSG00000101846 | STS | X | F |  | F |  |  |  | F |
| ENSG00000114374 | USP9Y | Y |  | M | M | M | M | M | M |
| ENSG00000183878 | UTY | Y |  | M | M | M | M | M | M |
| ENSG00000005889 | ZFX | X |  | F | F | F | F | F | F |
| ENSG00000067646 | ZFY | Y |  | M | M | M | M | M | M |

CVS = chorionic villus sampling tissue (late first trimester)

SCT = syncytiotrophoblast (epithelial)

CTB = cytotrophoblast (epithelial)

AEC = arterial endothelial cells (endothelial)

VEC = venous endothelial cells (endothelial)

**References**

1. Cvitic S, Longtine MS, Hackl H, Wagner K, Nelson MD, Desoye G, Hiden U. The Human Placental Sexome Differs between Trophoblast Epithelium and Villous Vessel Endothelium. PLoS ONE. 2013;8(10):e79233.
