# Supplementary figures and images for: Sex differences in the late first trimester human placenta transcriptome
Source: Biol Sex Differ. 2018 Jan 15;9:4. doi: 10.1186/s13293-018-0165-y (PMC5769539; doi:10.1186/s13293-018-0165-y)

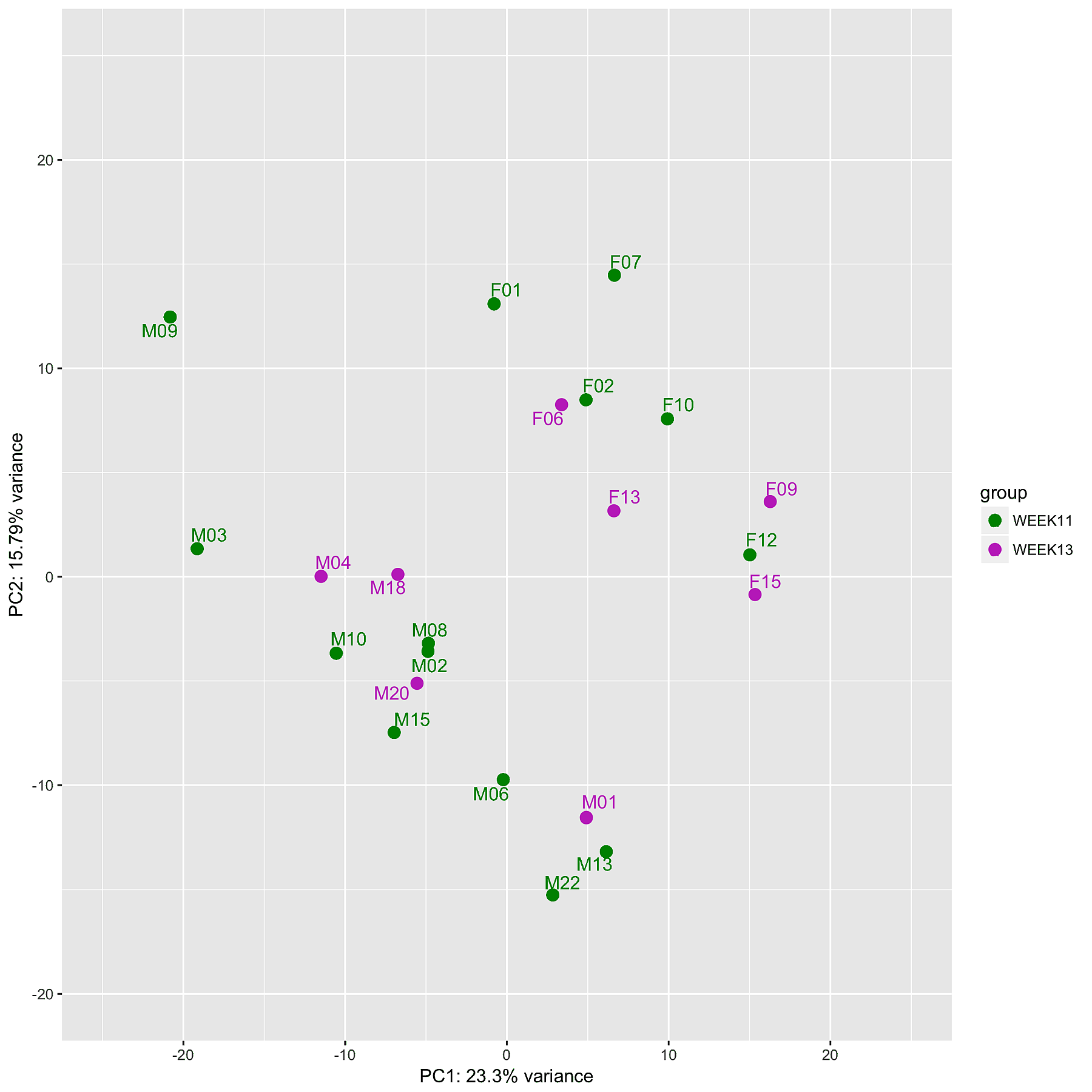

Supplement: Supplementary file 7 — Principal components analysis for week 11 vs week 13 subgroups. Early and late CVS collection time does not separate the samples. Green: week 11 samples. Purple: week 13 samples. (TIFF 610 kb) [file 13293_2018_165_MOESM7_ESM.tif]
