# Supplementary material for: Sex differences in the late first trimester human placenta transcriptome
Source: Biol Sex Differ. 2018 Jan 15;9:4. doi: 10.1186/s13293-018-0165-y (PMC5769539; doi:10.1186/s13293-018-0165-y)

**Additional file 9.** Week 13 DESeq2 subanalysis for sex differences.

**(A)** **Principal components analysis shows male and female clusters.** Blue: male samples (n=4). Red: female samples (n=4).


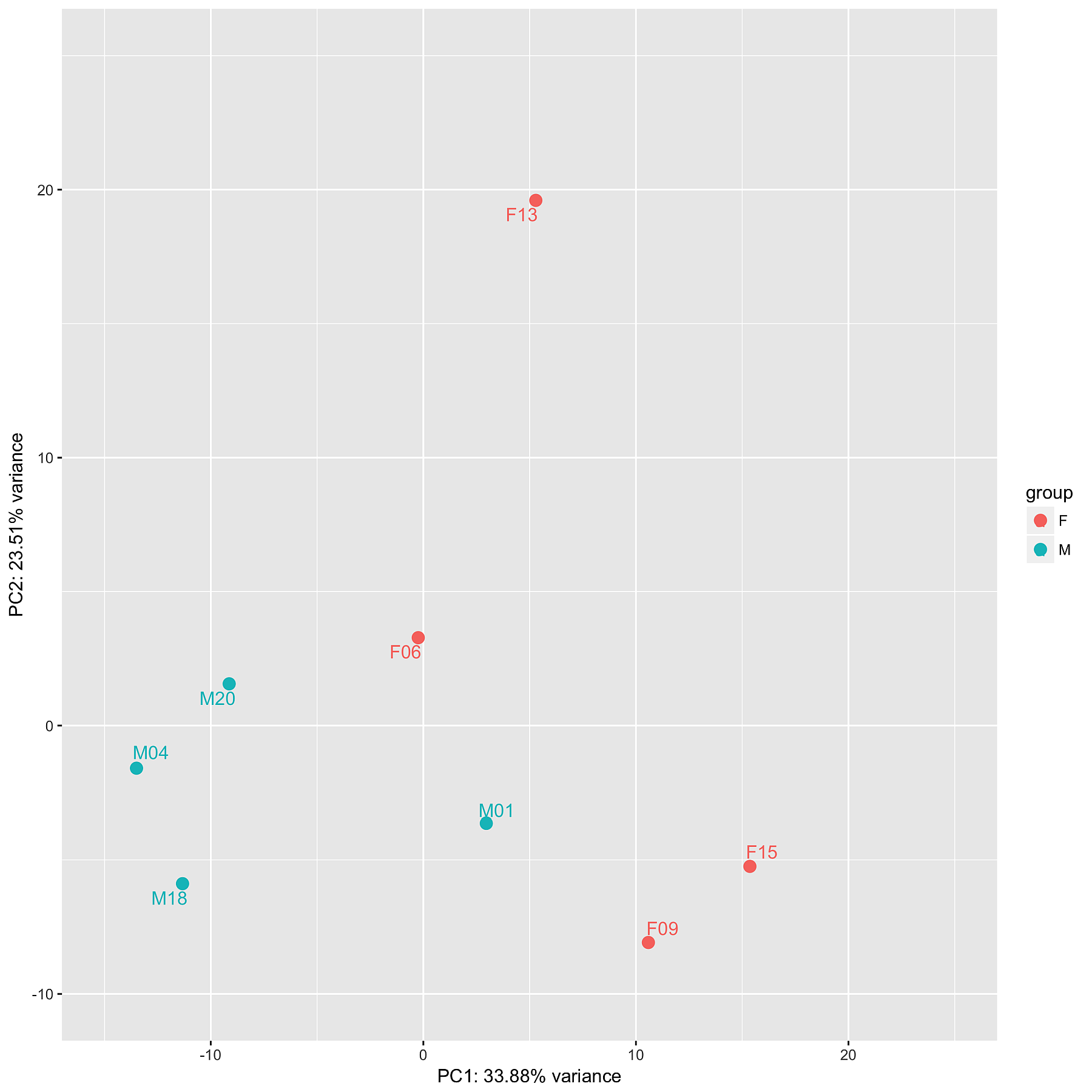


**(B)** **Volcano plot of DESeq2 results before FPKM cutoff.** Week 13 subanalysis. Volcano plot for all genes detected by RNA-seq, without an FPKM cutoff for expression. Red points: genes significantly different between sexes (Benjamini-Hochberg procedure to control for false discovery rate, FDR<0.05). Orange points: genes with 2-fold change or greater, |LogFC|>1 where FC = female/male fold-change. No genes fit criteria for orange points. Blue points: genes which are both significantly different (FDR<0.05) and have a 2-fold change or greater difference in expression between males and females. Black points: other genes.


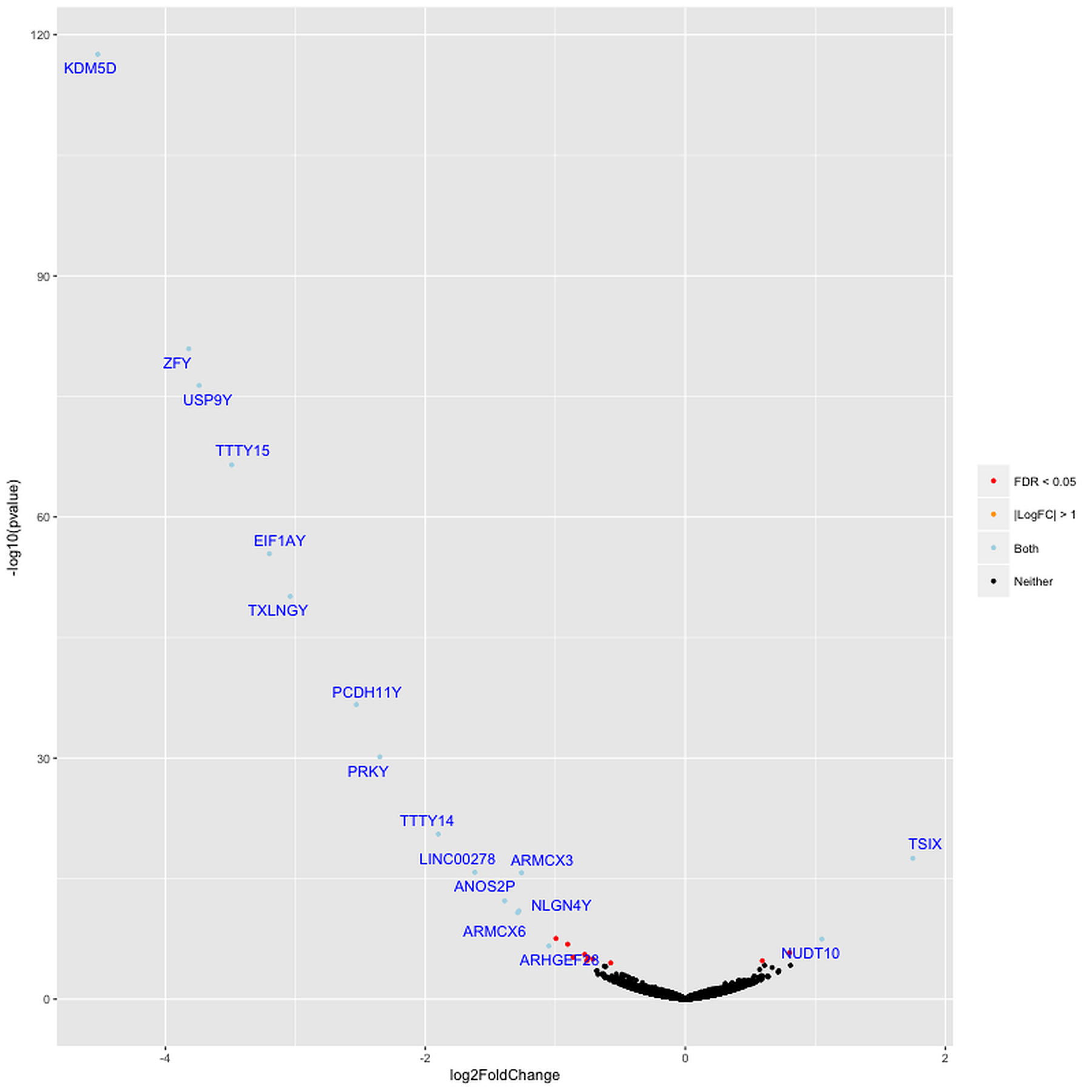


**(C) MA plot of DESeq2 results before FPKM cutoff.** Week 13 subanalysis. MA plot shows log2 of fold-changes (female/male) versus baseMean expression values (gene counts normalized by sequencing depth). Red points: genes significantly different between sexes (Benjamini-Hochberg procedure to control for false discovery rate, FDR<0.05). Black points: other genes.


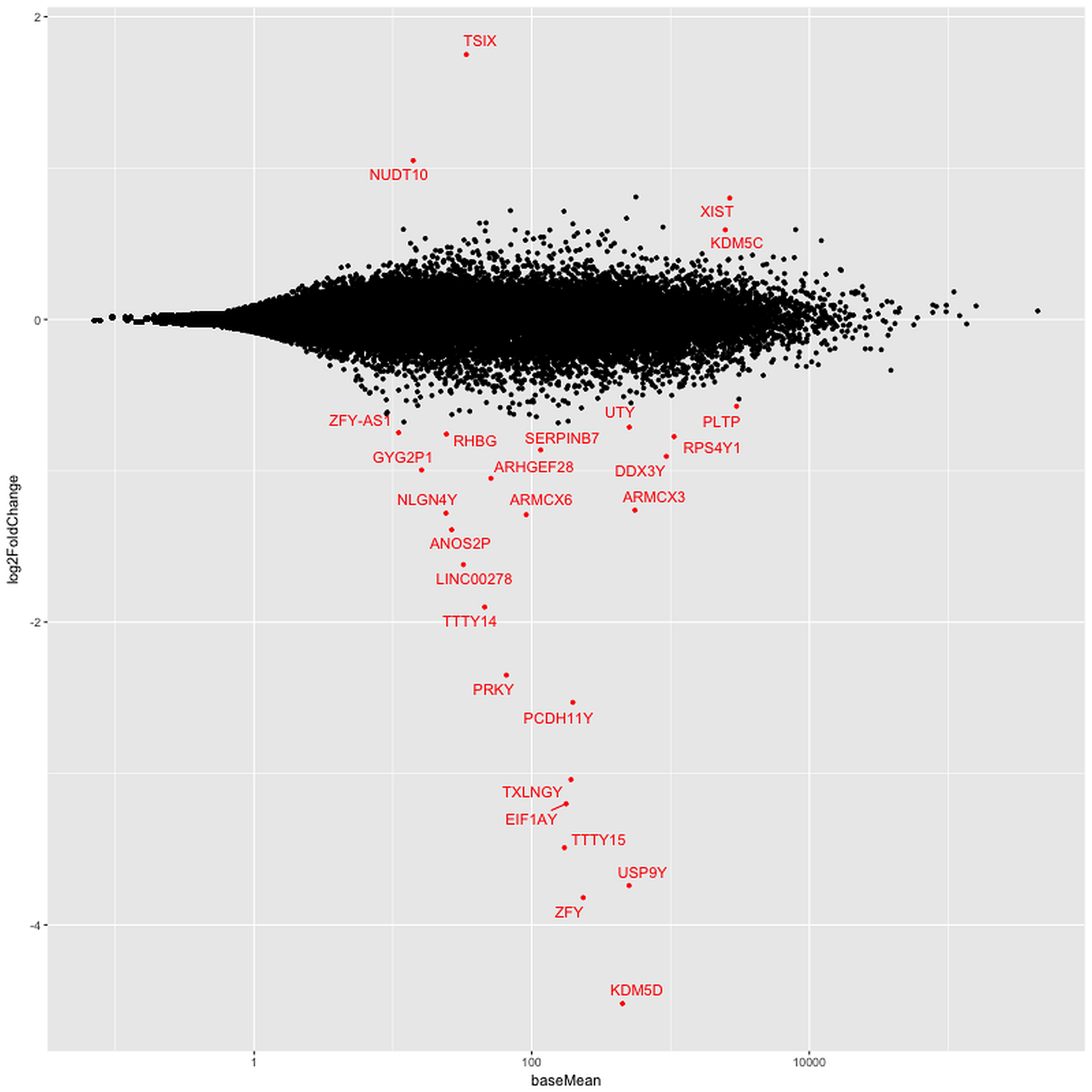

Supplement: Supplementary file 9 — Week 13 DESeq2 subanalysis for sex differences. a Principal components analysis for week 13 subgroup shows male and female clusters. b Volcano plot before FPKM cutoff. c MA plot before FPKM cutoff. (DOC 863 kb) [file 13293_2018_165_MOESM9_ESM.doc]
